# Adaptive Estimation and Cooperative Guidance for Active Aircraft Defense in Stochastic Scenario

**DOI:** 10.3390/s19040979

**Published:** 2019-02-25

**Authors:** Feng Fang, Yuanli Cai, Zhenhua Yu

**Affiliations:** 1 School of Electronic and Information Engineering, Xi’an Jiaotong University, Xi’an 710049, China; arrowfang@stu.xjtu.edu.cn; 2 School of Information and Navigation, Air Force Engineering University, Xi’an 710077, China; zhenhua_yu@163.com

**Keywords:** adaptive cooperative guidance, multiple model adaptive estimator, square-root cubature Kalman filter, estimation enhancement, active defense

## Abstract

The active aircraft defense problem is investigated for the stochastic scenario wherein a defending missile (or a defender) is employed to protect a target aircraft from an attacking missile whose pursuit guidance strategy is unknown. For the purpose of identifying the guidance strategy, the static multiple model estimator (sMME) based on the square-root cubature Kalman filter is proposed, and each model represents a potential attacking missile guidance strategy. Furthermore, an estimation enhancement approach is provided by using pseudo-measurement. For each model in the sMME, the model-matched cooperative guidance laws for the target and defender are derived by formulating the active defense problem as a constrained linear quadratic problem, where an accurate defensive interception and the minimum evasion miss distance are both considered. The proposed adaptive cooperative guidance laws are the result of mixing the model-matched optimal cooperative guidance laws in the criterion of maximum a posteriori probability in the framework of the sMME. By adopting the adaptive cooperative guidance laws, the target can facilitate the defender’s interception with the attacking missile with less control effort. Also, simulation results show that the proposed guidance laws increase the probability of successful target protection in the stochastic scenario compared with other defensive guidance laws.

## 1. Introduction

With the development of advanced pursuit guidance laws, an attacking missile can intercept a low-maneuverability target accurately. In order to protect the target, a widely discussed topic in recent years is the active defense countermeasure, whereby a defending missile (or a defender) is launched from the target or a target-friendly platform to intercept the attacking missile. There are several approaches to investigating the active defense problem, including optimal control [1,2,3,4,5], differential game [6,7,8,9,10], sliding mode control [11], and line-of-sight guidance [12,13]. In [1,2,3,4,5], the authors used optimal control theory to derive cooperative guidance laws for target and defender with the assumption that the pursuit guidance law of an attacking missile is fixed and known. In [6,7,8,9,10], the authors adopted differential game theory to analyze the dynamic conflicts and design the associated guidance laws. In [11], a cooperative guidance law based on sliding model control was proposed, and its objective was to make the zero-effort miss distance and zero-effort relative velocity of missile–defender engagement both zero. In [12,13], the main idea was the use of line-of-sight guidance to ensure that the defender remains on the line joining the target and attacking missile, and thus, the defender will block the attacking missile.

In all of the above work, the active aircraft defense was discussed for deterministic scenarios, where the maneuvers and state information of each aircraft are exactly known and directly used to calculate the guidance commands. However, in practical applications, the guidance strategy of enemy aircraft is unknown, and the state information needs to be estimated from noisy measurements. Therefore, it is necessary to add a preprocessed module (i.e., estimation module) to deal with the stochastic scenarios of measurement noise and unknown guidance strategy. Also, the performance of the closed loop of guidance and estimation needs further analysis. This is an important motivation for the present effort to investigate the combined estimation and guidance algorithm in the stochastic scenario.

In [14,15], multiple-model adaptive estimators were presented to identify the purist guidance strategy of a homing missile. In [14], a multiple-model adaptive evasion strategy that enables a target’s evasion of a homing missile was proposed, and in [15], cooperative multiple-model adaptive guidance for the defender and target was designed. Inspired by [14,15], adaptive cooperative guidance laws for the target and defender in the stochastic scenario are proposed; the proposed approach combines the sMME-SRCKF and model-matched optimal cooperative guidance laws. The sMME-SRCKF refers to the static multiple model estimator (sMME) that adopts the square-root cubature Kalman filter (SRCKF) as the model-matched nonlinear filter, and each model of the sMME-SRCKF represents a potential guidance law of the attacking missile. The output of the sMME-SRCKF includes a state estimate and model probability, where the former is used to calculate the model-matched cooperative guidance commands, and the latter is used to mix the model-matched guidance laws in the criterion of maximum a posteriori probability. Although the adaptive guidance laws in this paper and in Reference [15] are both designed using a similar approach, which combines an adaptive estimator and model-matched optimal cooperative guidance laws, they are very different. First, the most important difference is that the core of adaptive guidance laws, i.e., the model-matched cooperative guidance laws, are totally different. In [15], the authors designed the defender’s guidance law by using the known future maneuver of the protected target; in the case of a target using a bang–bang maneuver, the optimal switch time of this maneuver was solved to minimize the control effort of the defender. However, in this study, without knowing the information of the future target, the optimal cooperative guidance laws for the defender and target were derived together by solving a constrained linear quadratic problem. Second, the criterion to mix model-matched cooperative guidance laws in this paper is maximum a posteriori probability criterion. Compared with the use of the minimum mean-square-error criterion in [15], the criterion used in this study can reduce the computational burden. Third, the model-matched filter described in this paper is SRCKF, which has two advantages (see Section 3.1 and Section 5.3.1) compared with the extended Kalman filter (EKF) used as a model-matched filter in [15].

The main contribution of this work includes the following aspects. (i) The adaptive cooperative guidance laws can increase the probability of successful target protection in the stochastic active defense scenario, since it is designed to apply to the entire duration of active defense engagement rather than solely the defender–missile engagement, as in [1,2,3,4,15]. Thus, an additional chance for the target to evade the attacking missile is presented if the defender fails to intercept the attacking missile because it is negatively affected by stochastic factors; this issue demonstrated in Section 5.2. (ii) The model-matched cooperative guidance laws are designed to consider the two sufficient conditions of successful active defense, i.e., small defender–missile miss distance and minimum missile–target evasion distance. In [5], the authors also considered both of these conditions, but the two-dimensional generalized dead-zone functions require computation to generate guidance commands, which seems a little complex. On the other hand, the cooperative guidance laws in this paper are easily computed. (iii) Estimation enhancement is analyzed by using pseudo-measurement, and a guideline is provided for adjusting the location geometry of active defense to acquire good-quality estimation and then to improve further the performance of adaptive cooperative guidance laws.

This paper is organized as follows. In Section 2, the kinematic equations and estimation model of active aircraft defense are introduced. In Section 3, the sMME-SRCKF is proposed, and the estimation enhancement analysis is presented. The adaptive cooperative guidance laws for defender and target are derived in Section 4. The performance of the proposed guidance laws and filtering approach is analyzed in Section 5, and some remarkable conclusions are drawn in Section 6.

## 2. Preliminary

### 2.1. Kinematic Equations of Active Defense

**Figure 1:**
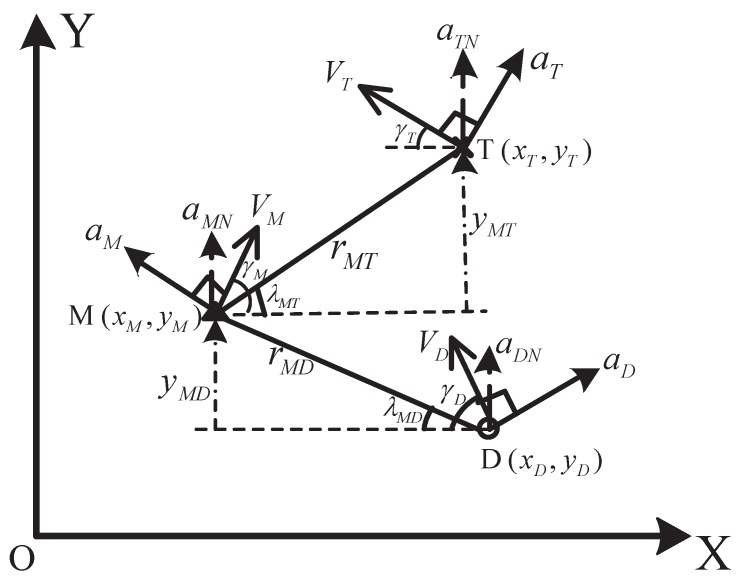
The geometry of active aircraft defense.

The active aircraft defense contains three aircraft: an attacking missile denoted as M, an evading target denoted as T, and a defender denoted as D. The attacking missile uses an interception guidance law to pursue the evading target. The defender, which is launched from the target or a target-friendly platform, tries to kill the attacking missile before it intercepts the target. The active aircraft defense can be divided into two engagements, which are defender–missile (D–M) engagement and missile–target (M–T) engagement. The geometry of active aircraft defense is shown in Figure 1. The *X*-axis is selected as the initial line-of-sight (LOS) of M–T engagement, and the *Y*-axis is normal to the *X*-axis. The subscripts *T*, *D*, and *M* represent the target, defender, and attacking missile, respectively. $\left(x_{i},y_{i}\right)$, $V_{i}$, $a_{i}$, and $\gamma_{i}$, where i=T,D,M, represent the position, velocity, acceleration, and flight-path angle of each aircraft, and $a_{i}$ is normal to its velocity. $a_{iN}$ is the acceleration component along the *Y*-axis, and it satisfies $a_{iN}=a_{i}\cos\gamma_{i}$, where i=T,D,M. $\lambda_{MT}$ and $\lambda_{MD}$ are the LOS angles of M–T and D–M engagements; $r_{MT}$ and $r_{MD}$ are the relative distances of M–T and D–M; and $y_{MT}$ and $y_{MD}$ are the relative displacements along the *Y*-axis.

In this paper, there are three assumptions: (i) The M–T and D–M engagements occur around the triangle-collision courses, so both types of engagements can be linearized around the initial LOS of M–T (or the *X*-axis). This can be realized at the endgame of engagement, since most of the guidance error has been removed after the midcourse guidance. (ii) The set of the attacking missile’s guidance laws contains proportional navigation (PN), augmented proportional navigation (APN), and optimal guidance law (OGL) (shown in Equations (3) and (4)); the attacking missile uses one of them to pursue the target, and it is unknown to the defender–target team. (iii) The aircraft’s dynamics is represented by arbitrary-order linear equations [1]:

(1) $$\begin{array}{c} {\dot{q}}_{i}=A_{i}q_{i}+b_{i}u_{iN},\;i=T,D,M\\ a_{iN}=C_{i}q_{i}+d_{i}u_{iN},\;i=T,D,M \end{array}$$

where $q_{i}$ represents the internal state vector of the aircraft with the dimension $\dim\left(q_{i}\right)=n_{i}$ for i=T,D,M; and $u_{iN}$ is the control command component along the *Y*-axis and satisfies $u_{iN}=u_{i}\cos\gamma_{i}$, where $u_{i}$ is the total control command perpendicular to the velocity. If an aircraft has first-order dynamics with time constant $\tau_{i}$, then we have $A_{i}=-1/\tau_{i}$, $b_{i}=1/\tau_{i}$, $C_{i}=1$, and $d_{i}=0$. If an aircraft has ideal dynamics, then we have $A_{i}=0$, $b_{i}=0$, $C_{i}=0$, and $d_{i}=1$. This means that the acceleration is equal to the guidance command, which can be obtained immediately without delay. For example, if an aircraft uses the thrust vector control engine, then the acceleration dynamics is nearly ideal.

On the basis of the linearization assumption, the D–M interception time $t_{f_{MD}}$ and M–T interception time $t_{f_{MT}}$ are approximately calculated as

(2) $$\begin{array}{c} t_{f_{MD}}≃\frac{r_{MD0}}{V_{M0}\cos\left(\gamma_{M0}+\lambda_{MD0}\right)+V_{D0}\cos\left(\gamma_{D0}-\lambda_{MD0}\right)}\\ t_{f_{MT}}≃\frac{r_{MT0}}{V_{M0}\cos\left(\gamma_{M0}-\lambda_{MT0}\right)+V_{T0}\cos\left(\gamma_{T0}+\lambda_{MT0}\right)} \end{array}$$

where the subscript 0 indicates the initial instant.

According to [1], under the linearization assumption, the traditional attacking missile’s guidance laws can be written in the general form of Equation (3), which is a function of the M–T engagement’s relative variables and possibly the control of the target.

(3) $$u_{MN}=\left[k_{1},k_{2},k_{M},k_{T}\right]{\left[y_{MT},{\dot{y}}_{MT},q_{M}^{T},q_{T}^{T}\right]}^{T}+k_{u_{T}}u_{TN}$$

The pursuit guidance laws of PN, APN, and OGL have the following forms:

(4) $$\left\{\begin{array}{c} \begin{array}{c} \mathrm{PN}:k_{1}=\frac{N_{\mathrm{PN}}}{t_{go_{MT}}^{2}};\;k_{2}=\frac{N_{\mathrm{PN}}}{t_{go_{MT}}};\;k_{M}={\left[0\right]}_{1\times n_{M}};k_{T}={\left[0\right]}_{1\times n_{T}};\;k_{u_{T}}=0.\\ \mathrm{APN}:k_{1}=\frac{N_{\mathrm{APN}}}{t_{go_{MT}}^{2}};\;k_{2}=\frac{N_{\mathrm{APN}}}{t_{go_{MT}}};\;k_{M}={\left[0\right]}_{1\times n_{M}};k_{T}=\frac{N_{\mathrm{APN}}C_{T}}{2};\;k_{u_{T}}=\frac{N_{\mathrm{APN}}d_{T}}{2}.\\ \mathrm{OGL}:k_{1}=\frac{N_{\mathrm{OGL}}}{t_{go_{MT}}^{2}};\;k_{2}=\frac{N_{\mathrm{OGL}}}{t_{go_{MT}}};\;k_{M}=\frac{-N_{\mathrm{OGL}}\psi \left(\theta_{MT}\right)C_{M}}{\theta_{MT}^{2}};k_{T}=\frac{N_{\mathrm{OGL}}C_{T}}{2};\;k_{u_{T}}=\frac{N_{\mathrm{OGL}}d_{T}}{2}. \end{array} \end{array}\right.$$

where $N_{j}$, with j={PN,APN,OGL} as the navigation gains; $t_{go_{MT}}$ is the time-to-go of M–T engagement, i.e., $t_{go_{MT}}=t_{f_{MT}}-t$; and $\psi \left(\theta_{MT}\right)=e^{-\theta_{MT}}+\theta_{MT}-1$, with $\theta_{MT}=t_{go_{MT}}/\tau_{M}$. $N_{\mathrm{PN}}$ and $N_{\mathrm{APN}}$ are constants between 3 and 5, and $N_{\mathrm{OGL}}$ satisfies

(5) $$N_{\mathrm{OGL}}=\frac{6\theta_{MT}^{2}\psi \left(\theta_{MT}\right)}{3+6\theta_{MT}-6\theta_{MT}^{2}+2\theta_{MT}^{3}-3e^{-2\theta_{MT}}-12\theta_{MT}e^{-\theta_{MT}}}$$

Defining the state vector of active defense as

(6) $$X={\left[y_{MT},{\dot{y}}_{MT},q_{M}^{T},q_{T}^{T},y_{MD},{\dot{y}}_{MD},q_{D}^{T}\right]}^{T}$$

and using the attacking missile’s guidance law in Equation (3), the kinematic equation of active defense is

(7) $$\dot{X}=A\left(t\right)X+B_{T}\left(t\right)u_{TN}+B_{D}\left(t\right)u_{DN}$$

where

(8) $$A\left(t\right)=\left[\begin{array}{cc} A_{11}\left(t\right) & A_{12}\left(t\right)\\ A_{21}\left(t\right) & A_{22}\left(t\right) \end{array}\right]$$

(9) $$\begin{array}{c} A_{11}\left(t\right)=\left[\begin{array}{cccc} 0 & 1 & {\left[0\right]}_{1\times n_{M}} & {\left[0\right]}_{1\times n_{T}}\\ -d_{M}k_{1} & -d_{M}k_{2} & -C_{M}-d_{M}k_{M} & C_{T}-d_{M}k_{T}\\ b_{M}k_{1} & b_{M}k_{2} & A_{M}+b_{M}k_{M} & b_{M}k_{T}\\ {0]}_{n_{T}\times 1} & {\left[0\right]}_{n_{T}\times 1} & {\left[0\right]}_{n_{T}\times n_{M}} & A_{T} \end{array}\right],\;A_{12}\left(t\right)={\left[0\right]}_{\left(n_{M}+n_{T}+2\right)\times \left(n_{D}+2\right)}\\ A_{21}\left(t\right)=\left[\begin{array}{cccc} 0 & 0 & {\left[0\right]}_{1\times n_{M}} & {\left[0\right]}_{1\times n_{T}}\\ d_{M}k_{1} & d_{M}k_{2} & C_{M}+d_{M}k_{M} & d_{M}k_{T}\\ {0]}_{n_{D}\times 1} & {\left[0\right]}_{n_{D}\times 1} & {\left[0\right]}_{n_{D}\times n_{M}} & {\left[0\right]}_{n_{D}\times n_{T}} \end{array}\right],\;A_{22}\left(t\right)=\left[\begin{array}{ccc} 0 & 1 & {\left[0\right]}_{1\times n_{D}}\\ 0 & 0 & -C_{D}\\ {0]}_{n_{D}\times 1} & {\left[0\right]}_{n_{D}\times 1} & A_{D} \end{array}\right] \end{array}$$

and

(10) $$B_{T}\left(t\right)=\left[\begin{array}{c} 0\\ d_{T}-d_{M}k_{u_{T}}\\ b_{M}k_{u_{T}}\\ b_{T}\\ 0\\ d_{M}k_{u_{T}}\\ {0]}_{n_{D}\times 1} \end{array}\right],\;B_{D}\left(t\right)=\left[\begin{array}{c} {\left[0\right]}_{(n_{M}+n_{T}+3)\times 1}\\ -d_{D}\\ b_{D} \end{array}\right]$$

### 2.2. Estimation Model

According to Figure 1, the kinematic equation of the attacking missile is described as

(11) $$\left\{\begin{array}{c} {\dot{x}}_{M}=V_{M}\cos\gamma_{M}\\ {\dot{y}}_{M}=V_{M}\sin\gamma_{M}\\ {\dot{\gamma }}_{M}=\frac{C_{M}q_{M}+d_{M}u_{MN}}{V_{M}\cos\gamma_{M}}\\ {\dot{q}}_{M}=A_{M}q_{M}+b_{M}u_{MN}\\ {\dot{V}}_{M}=0 \end{array}\right.$$

Defining the state vector of the attacking missile as $X_{M}=[x_{M},y_{M},\gamma_{M},q_{M}^{T},V_{M}{]}^{T}$, Equation (11) can be rewritten as

(12) $${\dot{X}}_{M}=f\left(X_{M},u_{MN}\right)$$

Assuming the defender and target track the attacking missile with infrared radar, the measurement model of active defense during D–M engagement (i.e., $t\in [0,\;t_{f_{MD}}]$) is

(13) $$z\left(k\right)=\left[\begin{array}{c} z_{MT}\left(k\right)\\ z_{MD}\left(k\right) \end{array}\right]=h\left(X_{M}\left(k\right)\right)+\upsilon \left(k\right)=\left[\begin{array}{c} \arctan\left(\frac{y_{T}-y_{M}}{x_{T}-x_{M}}\right)\\ \arctan\left(\frac{y_{M}-y_{D}}{x_{D}-y_{M}}\right) \end{array}\right]+\left[\begin{array}{c} \upsilon_{MT}\left(k\right)\\ \upsilon_{MD}\left(k\right) \end{array}\right]$$

where $z\left(k\right)={\left[z_{MT}\left(k\right),\;z_{MD}\left(k\right)\right]}^{T}$ represents the measurements of M–T and D–M LOS angles, and $\upsilon \left(k\right)={\left[\upsilon_{MT}\left(k\right),\upsilon_{MD}\left(k\right)\right]}^{T}$ is a mutually independent zero-mean white Gaussian noise. The covariance matrix of measurement noise is

(14) $$R\left(k\right)=\left[\begin{array}{cc} \sigma_{MT}^{2}\left(k\right) & 0\\ 0 & \sigma_{MD}^{2}\left(k\right) \end{array}\right]$$

where $\sigma_{MT}^{2}\left(k\right)$ and $\sigma_{MD}^{2}\left(k\right)$ are the variances of $\upsilon_{MT}\left(k\right)$ and $\upsilon_{MD}\left(k\right)$, respectively.

After the missile–defender engagement terminates (i.e., $t\in (t_{f_{MD}},\;t_{f_{MT}}]$), only the target uses its sensor to track the attacking missile, and then the measurement model becomes

(15) $$z\left(k\right)=z_{MT}\left(k\right)=\arctan\left(\frac{y_{T}-y_{M}}{x_{T}-x_{M}}\right)+\upsilon_{MT}\left(k\right)$$

Thus, the estimation model is built by using the process model of Equation (11) and the measurement model of Equation (13) or (15), and then the nonlinear Kalman filter can be used to estimate the state of the attacking missile.

## 3. Guidance Identification and State Estimation

### 3.1. Static Multiple Model Estimator

In this subsection, a method called the sMME-SRCKF is proposed to deal with the scenario of an unknown guidance strategy of an attacking missile. The sMME, also known as the multiple-model adaptive estimator [16], is used to identify the guidance law; meanwhile, the square-root cubature Kalman filter (SRCKF) is employed as the model-matched filter. The SRCKF is a square-root version of the cubature Kalman filter, which is proved as a good nonlinear Kalman filter having good numerical stability, low computational complexity, and satisfactory filtering accuracy as compared with other methods [17,18,19]. Because of its good performance, the improved estimation algorithms based on the cubature Kalman filter are widely used in target tracking and navigation systems [20,21,22]. Thus, the SRCKF is adopted as the model-matched filter. The sMME addresses a set of the potential models of the system, and then the model-matched SRCKF is set up to yield model-conditioned state estimate and error covariance. Assuming that there are *N* potential attacking missile guidance laws, the guidance commands are defined as $u_{MN}^{j}$, with j=1,2,⋯,N. Then, by substituting $u_{MN}=u_{MN}^{j}$ into Equation (11), *N* estimation models are obtained on the basis of the process model shown in Equation (11) and the measurement model shown in Equation (13) or (15). On the basis of these estimation models, the algorithm of the sMME-SRCKF is described in the following three steps.

*Step 1*: Run *N* parallel SRCKFs to yield each model-conditioned state estimate and error covariance, namely, ${\hat{X}}_{M}^{j}\left(k|k\right)$ and $P^{j}\left(k|k\right)$ (see Equations (A11) and (A15)). The original work of the SRCKF is introduced in [17]. However, the filtering problem in this section is a little different from the one in [17], since the process equation (shown in Equation (11)) is a differential equation rather than a difference equation, as in [17]. Thus, the evaluation of the propagated cubature points in the time update needs revising. In other words, according to Equation (11), we use the fourth-order Runge–Kutta method to calculate the integration to obtain the propagated cubature points. The details of using the SRCKF to estimate the attacking missile’s state information are given in Appendix A.

*Step 2*: Model probability update. The *j*th model probability $\mu^{j}\left(k\right)$ is obtained according to the Bayes’ formula, which is

(16) $$\mu^{j}\left(k\right)=\frac{\Lambda^{j}\left(k\right)\mu^{j}\left(k-1\right)}{\sum_{i=1}^{N}\Lambda^{i}\left(k\right)\mu^{i}\left(k-1\right)}$$

where $\Lambda^{j}\left(k\right)$ is the *j*th model-conditioned likelihood function, computed as

(17) $$\Lambda^{j}\left(k\right)=\frac{\exp[-0.5{\left({\tilde{z}}^{j}\left(k|k\right)\right)}^{T}{\left(P_{zz}^{j}\left(k|k-1\right)\right)}^{-1}\left({\tilde{z}}^{j}\left(k|k\right)\right)]}{\sqrt{{\left(2\pi \right)}^{n}\left|P_{zz}^{j}\left(k|k-1\right)\right|}}$$

where ${\tilde{z}}^{j}\left(k|k\right)$ and $P_{zz}^{j}\left(k|k-1\right)$ are the *j*th model-conditioned innovation and innovation covariance (see Equation (A10)).

*Step 3*: Update the state estimate (i.e., ${\hat{X}}_{M}\left(k|k\right)$) and error covariance (i.e., P(k|k)) by combining the model-conditioned state estimate and error covariance, as shown in Equations (18) and (19).

(18) $${\hat{X}}_{M}\left(k|k\right)=\sum_{j=1}^{N}\mu^{j}\left(k\right){\hat{X}}_{M}^{j}\left(k|k\right)$$

(19) $$P\left(k|k\right)=\sum_{j=1}^{N}\mu^{j}\left(k\right)\left\{P^{j}\left(k|k\right)+\left[{\hat{X}}_{M}^{j}\left(k|k\right)-{\hat{X}}_{M}\left(k|k\right)\right]{\left[{\hat{X}}_{M}^{j}\left(k|k\right)-{\hat{X}}_{M}\left(k|k\right)\right]}^{T}\right\}$$

**Figure 2:**
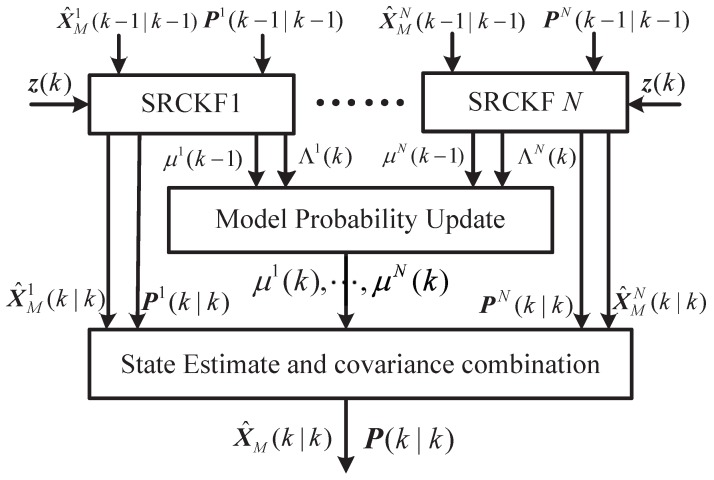
The structure of adaptive cooperative guidance laws.

According to the above steps of the sMME-SRCKF, the schematic structure of the sMME-SRCKF is shown in Figure 2, where SRCKF1 to SRCKF *N* represent *N* parallel Kalman filters.

There are two reasons for adopting the SRCKF to estimate the state of the attacking missile. First, the SRCKF is a more accurate nonlinear filter than the traditional extended Kalman filter (EKF) and unscented Kalman filter (UKF) [17,18,19]. Second, by using the EKF in [15], the authors needed to compute the complex Jacobin matrix of Equation (12), defined as $F_{x}^{j}=\frac{\partial f(X_{M},u_{MN}^{j})}{\partial X_{M}}$, for the step of state prediction in each model. However, the derivation of the Jacobin matrix is complex, since $u_{MN}^{j}$ is a function of $X_{M}$. Additionally, the authors needed to compute the transition matrix on the basis of $F_{x}^{j}$, i.e., $\Phi_{M}\left(k,k-1\right)=e^{F_{x}^{j}T}$, and it is a little hard to calculate. However, the SRCKF adopted here is derivative-free for undesirable Jacobians and the transition matrix, and we only need to compute the cubature points in the state prediction (see Equations (A1) and (A2)), which is easier to calculate. Comparisons between the performance of SRCKF and EKF and between the performance of sMME-SRCKF and sMME-EKF are presented in Section 5.3.1.

### 3.2. Estimation Enhancement Analysis

In this subsection, the method of enhancing the estimation performance is discussed. The estimation module is processed before the guidance module, and the state estimate is used for computing the adaptive guidance laws (see Section 4.2). Thus, the estimation results have a great impact on the performance of adaptive cooperative guidance. For example, if the sMME-SRCKF can identify the exact guidance strategy of the attacking missile as soon as possible, then the command error of adaptive cooperative guidance will be reduced. Otherwise, a part of the target and defender’s control effort will be wasted as a result of the uncertain strategy of the attacking missile. Also, the more accurate the estimation, the larger the probability that the defender–target team is successful. For this purpose, we analyze the influence of the active defense location geometry on the estimation performance and then use it as a guideline to improve the estimation performance.

The analysis of estimation enhancement is based on the concept of pseudo-measurement described in [23]. According to Figure 1, the position of the attacking missile can be calculated by using the noisy measurements as

(20) $${\overset{⌢}{x}}_{M}=x_{D}-\frac{r_{DT}\cos\left(z_{MT}-\theta_{DT}\right)\cos\left(z_{MD}\right)}{\sin(z_{MT}+z_{MD})},\;{\overset{⌢}{y}}_{M}=y_{D}+\frac{r_{DT}\cos\left(z_{MT}-\theta_{DT}\right)\sin\left(z_{MD}\right)}{\sin(z_{MT}+z_{MD})}$$

where

(21) $$r_{DT}=\sqrt{{\left(x_{D}-x_{T}\right)}^{2}+{\left(y_{D}-y_{T}\right)}^{2}},\;\theta_{DT}=\arctan\left(\frac{x_{D}-x_{T}}{y_{T}-y_{D}}\right)$$

According to the measurement model of Equation (13), we have $z_{MT}=\lambda_{MT}+\upsilon_{MT}$ and $z_{MD}=\lambda_{MD}+\upsilon_{MD}$. Since $\upsilon_{MT}$ and $\upsilon_{MD}$ are small, Equation (21) can be linearized around $\lambda_{MT}$ and $\lambda_{MD}$ as

(22) $$\left\{\begin{array}{c} \begin{array}{cc} {\overset{⌢}{x}}_{M} & ≃x_{M}+\frac{r_{DT}\cos\left(\lambda_{MD}+\theta_{DT}\right)\cos\left(\lambda_{MD}\right)}{\sin^{2}\left(\lambda_{MT}+\lambda_{MD}\right)}\upsilon_{MT}+\frac{r_{DT}\cos\left(\lambda_{MT}-\theta_{DT}\right)\cos\left(\lambda_{MT}\right)}{\sin^{2}\left(\lambda_{MT}+\lambda_{MD}\right)}\upsilon_{MD}\\ {\overset{⌢}{y}}_{M} & ≃y_{M}-\frac{r_{DT}\cos\left(\lambda_{MD}+\theta_{DT}\right)\sin\left(\lambda_{MD}\right)}{\sin^{2}\left(\lambda_{MT}+\lambda_{MD}\right)}\upsilon_{MT}+\frac{r_{DT}\cos\left(\lambda_{MT}-\theta_{DT}\right)\sin\left(\lambda_{MT}\right)}{\sin^{2}\left(\lambda_{MT}+\lambda_{MD}\right)}\upsilon_{MD} \end{array} \end{array}\right.$$

where $x_{M}$ and $y_{M}$ are the true values of the position and are defined as

(23) $$x_{M}=x_{D}-\frac{r_{DT}\cos\left(\lambda_{MT}-\theta_{DT}\right)\cos\left(\lambda_{MD}\right)}{\sin(\lambda_{MT}+\lambda_{MD})},\;y_{M}=y_{D}+\frac{r_{DT}\cos\left(\lambda_{MT}-\theta_{DT}\right)\sin\left(\lambda_{MD}\right)}{\sin(\lambda_{MT}+\lambda_{MD})}$$

According to Equation (22), ${\overset{⌢}{x}}_{M}$ and ${\overset{⌢}{y}}_{M}$ can be viewed as pseudo-measurements at time step *k*, which has a non-stationary normal distribution, defined by

(24) $${\overset{⌢}{x}}_{M}∼N(x_{M},\sigma_{x_{M}}^{2}),\;{\overset{⌢}{y}}_{M}∼N(y_{M},\sigma_{y_{M}}^{2})$$

where

(25) $$\left\{\begin{array}{cc} \sigma_{x_{M}}^{2}= & \frac{r_{DT}^{2}\cos^{2}\left(\lambda_{MD}+\theta_{DT}\right)\cos^{2}\left(\lambda_{MD}\right)}{\sin^{4}\left(\lambda_{MT}+\lambda_{MD}\right)}\sigma_{MT}^{2}+\frac{r_{DT}^{2}\cos^{2}\left(\lambda_{MT}-\theta_{DT}\right)\cos^{2}\left(\lambda_{MT}\right)}{\sin^{4}\left(\lambda_{MT}+\lambda_{MD}\right)}\sigma_{MD}^{2}\\ \sigma_{y_{M}}^{2}= & \frac{r_{DT}^{2}\cos^{2}\left(\lambda_{MD}+\theta_{DT}\right)\sin^{2}\left(\lambda_{MD}\right)}{\sin^{4}\left(\lambda_{MT}+\lambda_{MD}\right)}\sigma_{MT}^{2}+\frac{r_{DT}^{2}\cos^{2}\left(\lambda_{MT}-\theta_{DT}\right)\sin^{2}\left(\lambda_{MT}\right)}{\sin^{4}\left(\lambda_{MT}+\lambda_{MD}\right)}\sigma_{MD}^{2} \end{array}\right.$$

According to Equation (25), if $\lambda_{MT}+\lambda_{MD}$ approaches zero, (i.e., the difference between the M–T LOS angle and the D–M LOS angle is small), then the variances of pseudo-measurement (i.e., $\sigma_{x_{M}}^{2}$ and $\sigma_{y_{M}}^{2}$) will increase significantly, and increased variance causes a deterioration in estimation accuracy, especially in the estimation of position. Thus, the estimation performance depends on the location geometry of the defender and target. In order to achieve a good-quality estimation, the difference between the M–T LOS angle and the D–M LOS angle should remain far from zero, which means that the trajectories of the defender and target should be separated clearly with respect to the attacking missile. This conclusion is used as a guideline for choosing an appropriate initial geometry of active defense to improve estimation performance, which is shown in Section 5.3.2.

## 4. Adaptive Cooperative Guidance Laws

### 4.1. Model-Matched Optimal Cooperative Guidance Laws

#### 4.1.1. Optimization Problem Formulation

For the identified guidance law of the attacking missile, the linearized kinematic equations are shown in Equations (7)–(10); on the basis of those, the optimal defensive guidance problem is formulated. The success of the defender–target team is defined as one of the following two sufficient conditions: (i) the D–M miss distance is small, and (ii) the M–T miss distance is larger than the lethal radius of the attacking missile. Here, both conditions are considered in designing cooperative guidance laws to achieve the largest probability of successful target protection, since the defender may fail to intercept the attacking missile due to its poor dynamics, acceleration saturation, or the negative effect of stochastic factors. For this purpose, a further target evasion maneuver is considered to increase the probability of successful active defense.

The objective function is defined as

(26) $$J=\frac{1}{2}\alpha y_{MD}^{2}\left(t_{f_{MD}}\right)+\frac{1}{2}\int_{0}^{t_{f_{MD}}}u_{DN}^{2}\left(\tau \right)d\tau +\frac{\beta }{2}\int_{0}^{t_{f_{MT}}}u_{TN}^{2}\left(\tau \right)d\tau$$

where $\left|y_{MD}\left(t_{f_{MD}}\right)\right|$ is the D–M miss distance, and α and β are positive penalty weights. The terminal constraint is defined as

(27) $$\left|y_{MT}\left(t_{f_{MT}}\right)\right|\geq \rho$$

where $\left|y_{MT}\left(t_{f_{MT}}\right)\right|$ is the M–T miss distance, and ρ is the expected evasion miss distance, which is larger than the lethal radius of the attacking missile. The guidance optimization problem is the minimization of the cost function in Equation (26) with the terminal constraint of Equation (27) based on the kinematic equation in Equation (7).

The M–T and D–M zero-effort miss distances, i.e., $\left|Z_{MD}\left(t\right)\right|$ and $\left|Z_{MT}\left(t\right)\right|$, are introduced to reduce the optimization problem’s order; they are defined as

(28) $$Z_{MT}\left(t\right)=D_{MT}\Phi \left(t_{f_{MT}},t\right)X\left(t\right),\;t\in \left[0,t_{f_{MT}}\right];\;Z_{MD}\left(t\right)=\left\{\begin{array}{c} D_{MD}\Phi \left(t_{f_{MD}},t\right)X\left(t\right),\;\;t\in \left[0,t_{f_{MD}}\right]\\ Z_{MD}\left(t_{f_{MD}}\right),\;\;\;\;t\in \left(t_{f_{MD}},t_{f_{MT}}\right] \end{array}\right.$$

where $D_{MD}=\left[0,0,{\left[0\right]}_{1\times n_{M}},{\left[0\right]}_{1\times n_{T}},1,0,{\left[0\right]}_{1\times n_{D}}\right]$, $D_{MT}=\left[1,0,{\left[0\right]}_{1\times n_{M}},{\left[0\right]}_{1\times n_{T}},0,0,{\left[0\right]}_{1\times n_{D}}\right]$ and Φ are the transition matrices associated with Equation (7). The physical meaning of $\left|Z_{MD}\left(t\right)\right|$ and $\left|Z_{MT}\left(t\right)\right|$ is the miss distance that the defender and target would achieve under the following condition: neither the defender nor the target would apply any control commands, while the attacking missile would still employ the guidance law from the current time instant to the final interception time. From Equation (28), we have $Z_{MD}\left(t_{f_{MD}}\right)=y_{MD}\left(t_{f_{MD}}\right)$ and $Z_{MT}\left(t_{f_{MT}}\right)=y_{MT}\left(t_{f_{MT}}\right)$. Since the defender’s guidance command only works in the D–M engagement, then $u_{DN}\left(t\right)\equiv 0$ for $t\in \left(t_{f_{MD}},t_{f_{MT}}\right]$ is obtained. Therefore, the cost function of Equation (26) can be rewritten as

(29) $$J=\frac{1}{2}\alpha Z_{MD}^{2}\left(t_{f_{MT}}\right)+\frac{1}{2}\int_{0}^{t_{f_{MT}}}u_{DN}^{2}\left(\tau \right)+\beta u_{TN}^{2}\left(\tau \right)d\tau$$

The derivatives of zero-effort miss distances with respect to time are

(30) $${\dot{Z}}_{MT}\left(t\right)=f_{T_{MT}}\left(t\right)u_{TN}\left(t\right),\;\;{\dot{Z}}_{MD}\left(t\right)=f_{T_{MD}}\left(t\right)u_{TN}\left(t\right)+f_{D_{MD}}\left(t\right)u_{DN}\left(t\right)$$

where

(31) $$f_{T_{MD}}\left(t\right)=D_{MD}\Phi_{etd}\left(t_{f_{MT}},t\right)B_{T},\;\;f_{D_{MD}}\left(t\right)=D_{MD}\Phi_{etd}\left(t_{f_{MT}},t\right)B_{D},\;\;f_{T_{MT}}\left(t\right)=D_{MT}\Phi \left(t_{f_{MT}},t\right)B_{T}$$

and $\Phi_{etd}\left(t_{f_{MT}},t\right)=\left\{\begin{array}{c} \Phi \left(t_{f_{MD}},t\right),\;\;t\leq t_{f_{MD}}\\ 0,\;\;\;\;\;t>t_{f_{MD}} \end{array}\right..$ The derivation of Equations (30) and (31) is shown in Appendix B.

On the basis of the dynamic model of Equation (30), the optimal guidance problem is order-reduced by using the cost function of Equation (29) with the terminal constraint as

(32) $$\left|Z_{MT}\left(t_{f_{MT}}\right)\right|\geq \rho$$

#### 4.1.2. Derivation of Optimal Cooperative Guidance Laws

Before solving the above optimal guidance problem, the following auxiliary optimization problem is considered: the terminal inequality constraint (i.e., Equation (32)) is replaced with the equality constraint of fixed M–T missile distance, i.e., $Z_{MT}\left(t_{f_{MT}}\right)=y_{f}$, where $y_{f}$ is an arbitrary real number satisfying $\left|y_{f}\right|\geq \rho$. The Hamilton of the auxiliary problem is

(33) $$\begin{array}{c} H=\frac{1}{2}u_{DN}^{2}+\frac{\beta }{2}u_{TN}^{2}+\lambda_{1}f_{T_{MT}}u_{TN}+\lambda_{2}f_{T_{MD}}u_{TN}+\lambda_{2}f_{D_{MD}}u_{DN} \end{array}$$

By applying $\frac{\partial H}{\partial u_{TN}}=0$ and $\frac{\partial H}{\partial u_{DN}}=0$ [24], the optimal control commands are obtained as

(34) $$u_{TN}^{o}\left(t\right)=\frac{-\lambda_{1}f_{T_{MT}}\left(t\right)-\lambda_{2}f_{T_{MD}}\left(t\right)}{\beta },\;\;u_{DN}^{o}\left(t\right)=-\lambda_{2}f_{D_{MD}}\left(t\right)$$

Using adjoint equation ${\dot{\lambda }}_{1}(t)=-\frac{\partial H}{\partial Z_{MT}\left(t\right)},\;{\dot{\lambda }}_{2}(t)=-\frac{\partial H}{\partial Z_{MD}\left(t\right)}$ and transversal condition $\left[\frac{\partial (0.5\alpha Z_{MD}^{2}\left(t\right))}{\partial Z_{MD}\left(t\right)}-\lambda_{2}\left(t\right)\right]{|}_{{}_{t=t_{f_{MT}}}}=0$, we have

(35) $$\lambda_{1}\left(t\right)=\mathrm{constant},\;\lambda_{2}\left(t\right)=\alpha Z_{MD}\left(t_{f_{MT}}\right)$$

Integrating Equation (30), the following equations are obtained:

(36) $$\left\{\begin{array}{c} \begin{array}{c} Z_{MT}\left(t_{f_{MT}}\right)=Z_{MT}\left(t\right)+\int_{t}^{t_{f_{MT}}}f_{T_{MT}}\left(\tau \right)u_{TN}^{o}\left(\tau \right)d\tau \\ Z_{MD}\left(t_{f_{MT}}\right)=Z_{MD}\left(t\right)+\int_{t}^{t_{f_{MT}}}f_{T_{MD}}\left(\tau \right)u_{TN}^{o}\left(\tau \right)+f_{D_{MD}}\left(\tau \right)u_{DN}^{o}\left(\tau \right)d\tau \end{array} \end{array}\right.$$

Substituting Equations (34) and (35) into Equation (36), $\lambda_{1}\left(t\right)$ and $\lambda_{2}\left(t\right)$ are determined as

(37) $$\lambda_{1}\left(t\right)=\frac{y_{f}-Z_{MT}\left(t\right)}{F_{1}}+\alpha \frac{F_{2}}{F_{1}}\frac{Z_{MD}\left(t\right)F_{1}+Z_{MT}\left(t\right)F_{2}-y_{f}F_{2}}{F_{1}-\alpha F_{1}F_{3}+\alpha F_{2}^{2}},\;\;\;\lambda_{2}\left(t\right)=\alpha \frac{Z_{MD}\left(t\right)F_{1}+Z_{MT}\left(t\right)F_{2}-y_{f}F_{2}}{F_{1}-\alpha F_{1}F_{3}+\alpha F_{2}^{2}}$$

where

(38) $$F_{1}=\int_{t}^{t_{f_{MT}}}-\frac{f_{T_{MT}}^{2}\left(\tau \right)}{\beta }d\tau ,\;\;F_{2}=\int_{t}^{t_{f_{MT}}}\frac{f_{T_{MT}}\left(\tau \right)f_{T_{MD}}\left(\tau \right)}{\beta }d\tau ,\;\;F_{3}=\int_{t}^{t_{f_{MT}}}-\frac{f_{T_{MD}}^{2}\left(\tau \right)}{\beta }-f_{D_{MD}}^{2}\left(\tau \right)d\tau$$

Thus, the optimal control commands of the auxiliary problem are determined by substituting Equations (37) and (38) into Equation (34).

Then, the optimal guidance problem can be solved by looking for the optimal value of $y_{f}$ in $\left|y_{f}\right|\geq \rho$ to minimize the cost function. Substituting Equations (34) and (37) into Equation (29), we can rewrite the cost function as

(39) $$J=\frac{\left(\alpha F_{1}F_{3}-F_{1}\right)}{L_{1}F_{1}}y_{f}^{2}+\frac{-2L_{2}}{L_{1}F_{1}}y_{f}+\frac{L_{3}^{2}F_{1}F_{3}-L_{2}^{2}+2L_{2}L_{3}F_{2}+(L_{3}^{2}F_{1}/\alpha^{2})}{L_{1}^{2}F_{1}}$$

where

(40) $$L_{1}=F_{1}-\alpha F_{1}F_{3}+\alpha F_{2}^{2},\;\;L_{2}=\left(\alpha F_{1}F_{3}-F_{1}\right)Z_{MT}\left(t\right)+\alpha F_{1}F_{2}Z_{MD}\left(t\right),\;\;L_{3}=\alpha F_{1}Z_{MD}\left(t\right)+\alpha F_{2}Z_{MT}\left(t\right)$$

According to Equation (38), we have $F_{1}<0$ and $F_{3}<0$. According to Equation (40), we have $L_{1}<0$, which is proved in Appendix C. Therefore, $\frac{\left(\alpha F_{1}F_{3}-F_{1}\right)}{L_{1}F_{1}}>0$ is obtained, and the optimal value $y_{f}^{*}$ is solved as

(41) $$y_{f}^{*}=z_{MT}^{*}\cdot \xi \left(\left|z_{MT}^{*}\right|-\rho \right)+\rho \cdot \mathrm{sign}\left(z_{MT}^{*}\right)\cdot \xi \left(\rho -\left|z_{MT}^{*}\right|\right)$$

where

(42) $$z_{MT}^{*}=Z_{MT}\left(t\right)+\frac{\alpha F_{1}F_{2}Z_{MD}\left(t\right)}{\alpha F_{1}F_{3}-F_{1}}$$

and

(43) $$\xi \left(\tau \right)=\left\{\begin{array}{cc} 1,\;\; & \tau >0\\ 1/2,\; & \tau =0\\ 0,\;\; & \tau <0 \end{array}\right.,\;\mathrm{sign}\left(\tau \right)=\left\{\begin{array}{cc} 1,\;\; & \tau \geq 0\\ -1,\;\; & \tau <0 \end{array}\right.$$

Replacing $y_{f}$ with $y_{f}^{*}$ in Equation (37) and then substituting it into Equation (34), the optimal cooperative guidance laws for the defender and the target are

(44) $$\left\{\begin{array}{c} u_{TN}^{*}\left(t\right)=-\frac{\left(\alpha f_{T_{MT}}\left(t\right)F_{1}F_{2}+\alpha f_{T_{MD}}\left(t\right)F_{1}^{2}\right)Z_{MD}\left(t\right)}{\beta F_{1}\left(F_{1}-\alpha F_{1}F_{3}+\alpha F_{2}^{2}\right)}+\frac{\left(f_{T_{MT}}\left(t\right)F_{1}-\alpha f_{T_{MT}}\left(t\right)F_{1}F_{3}-\alpha f_{T_{MD}}\left(t\right)F_{1}F_{2}\right)\left(Z_{MT}\left(t\right)-y_{f}^{*}\right)}{\beta F_{1}\left(F_{1}-\alpha F_{1}F_{3}+\alpha F_{2}^{2}\right)}\\ u_{DN}^{*}\left(t\right)=\frac{-\alpha f_{D_{MD}}\left(t\right)\left(Z_{MD}\left(t\right)F_{1}+Z_{MT}\left(t\right)F_{2}-y_{f}^{*}F_{2}\right)}{F_{1}-\alpha F_{1}F_{3}+\alpha F_{2}^{2}} \end{array}\right.$$

Here, a special case is considered, which is ρ=0. By substituting ρ=0 into Equation (41), the following equation is obtained as

(45) $$y_{f}^{*}=Z_{MT}\left(t\right)+\frac{\alpha F_{1}F_{2}Z_{MD}\left(t\right)}{\alpha F_{1}F_{3}-F_{1}}$$

Then, substituting Equation (45) into Equation (44), the optimal cooperative guidance laws become

(46) $$u_{TN}^{*}\left(t\right)=\frac{\alpha f_{T_{MD}}\left(t\right)Z_{MD}\left(t\right)}{\beta (\alpha F_{3}-1)},\;\;\;u_{DN}^{*}\left(t\right)=\frac{\alpha f_{D_{MD}}\left(t\right)Z_{MD}\left(t\right)}{\alpha F_{3}-1}$$

**Remark** **1.** *For the D–M engagement, the cooperative guidance laws in the special case of ρ=0 (i.e., Equation (46)) are equal to the cooperative guidance laws presented in [2] (see (53)∼(54) in [2]). This is because if ρ=0, the terminal constraint of Equation (32) is removed, and the optimization problem becomes the minimization of the cost function of Equation (26) on the basis of Kinematic Equations (7)–(10); this problem is identical to the one presented in [2]. Therefore, the cooperative guidance laws in this special case can be regarded as the guidance laws that consider only one sufficient condition of successful active defense, i.e., small D–M miss distance*.

#### 4.1.3. Target Evasion Guidance after Termination of Missile–Defender Engagement

After D–M engagement terminates, the active defensive engagement becomes M–T pursuit–evasion engagement. In this engagement, the flight time satisfies $t_{f_{MD}}<t\leq t_{f_{MT}}$. According to Equations (31) and (38), we have $f_{T_{MD}}\left(t\right)=0$, $f_{D_{MD}}\left(t\right)=0$, $F_{2}=0$ and $F_{3}=0$. Then, substituting them into Equation (44), the guidance command of the target can be written as

(47) $$u_{TN}^{*}=\left\{\begin{array}{cc} \frac{f_{T_{MT}}\left[Z_{MT}\left(t\right)-\rho \cdot \mathrm{sign}\left(Z_{MT}\left(t\right)\right)\right]}{\beta F_{1}}, & \left|Z_{MT}\left(t\right)\right|<\rho \\ 0, & \left|Z_{MT}\left(t\right)\right|\geq \rho \end{array}\right.$$

The guidance law in Equation (47) has the same form of the optimal evasion guidance law with a specific miss distance in [25] (see (20) in [25]). This is because after the D–M terminates, the optimization problem becomes one of minimizing the control effort of the target with the M–T miss distance constraint, and this is the same as the minimum-effort evasion guidance problem in [25]. If the attacking missile or pursuer uses the same pursuit guidance law shown in Equation (3), then both of the evasion guidance laws are same.

### 4.2. Adaptive Cooperative Guidance Laws

In Section 4.1, the model-matched optimal cooperative guidance laws are derived with perfect information. However, in the stochastic scenario, perfect information is unavailable. Thus, the estimated information of the filter is used to compute the guidance laws. In the sMME-SRCKF, the model-conditioned state estimation of the *j*th model can be obtained as ${\hat{X}}_{M}^{j}={\left[{\hat{x}}_{M}^{j},{\hat{y}}_{M}^{j},{\hat{\gamma }}_{M}^{j},{\left({\hat{q}}_{M}^{j}\right)}^{T},{\hat{V}}_{M}^{j}\right]}^{T}$, and it is used to calculate the estimated state of active defense as

(48) $${\hat{X}}^{j}={\left[{\hat{y}}_{MT}^{j},{\hat{\dot{y}}}_{MT}^{j},{\left({\hat{q}}_{M}^{j}\right)}^{T},q_{T}^{T},{\hat{y}}_{MD}^{j},{\hat{\dot{y}}}_{MD}^{j},q_{D}^{T}\right]}^{T}$$

where

(49) $${\hat{y}}_{MT}^{j}=y_{T}-{\hat{y}}_{M}^{j},\;\;{\hat{\dot{y}}}_{MT}^{j}=V_{T}\sin\gamma_{T}-{\hat{V}}_{M}^{j}\sin{\hat{\gamma }}_{M}^{j},\;\;{\hat{y}}_{MD}^{j}={\hat{y}}_{M}^{j}-y_{D},\;\;{\hat{\dot{y}}}_{MD}^{j}={\hat{V}}_{M}^{j}\sin{\hat{\gamma }}_{M}^{j}-V_{D}\sin\gamma_{D}$$

Then, the model-conditioned estimation of zero-effort miss distances is

(50) $${\hat{Z}}_{MT}^{j}\left(t\right)=D_{MT}\Phi \left(t_{f_{MT}},t\right){\hat{X}}^{j}\left(t\right),\;t\in \left[0,t_{f_{MT}}\right];\;{\hat{Z}}_{MD}^{j}\left(t\right)=\left\{\begin{array}{c} D_{MD}\Phi \left(t_{f_{MD}},t\right){\hat{X}}^{j}\left(t\right),\;\;t\in \left[0,t_{f_{MD}}\right]\\ {\hat{Z}}_{MD}^{j}\left(t_{f_{MD}}\right),\;\;\;\;\;t\in \left(t_{f_{MD}},t_{f_{MT}}\right] \end{array}\right.$$

The *j*th model-matched cooperative guidance laws (i.e., $u_{TN}^{*j}\left(k\right)$ and $u_{DN}^{*j}\left(k\right)$) are obtained by replacing $Z_{MD}\left(t\right)$ and $Z_{MT}\left(t\right)$ with ${\hat{Z}}_{MD}^{j}\left(t\right)$ and ${\hat{Z}}_{MT}^{j}\left(t\right)$ in Equation (44).

The adaptive cooperative guidance laws are derived by mixing the model-matched optimal guidance laws in the criterion of maximum a posteriori probability as

(51) $$u_{TN}^{A}\left(k\right)=u_{TN}^{*j}\left(k\right),\;\;u_{DN}^{A}\left(k\right)=u_{DN}^{*j}\left(k\right),\;\;j=\underset{i\in \{1,2,\cdots ,N\}}{\arg\max(\mu^{i}\left(k\right))}$$

Equation (51) shows that the model-matched cooperative guidance commands with the largest model probability are chosen as the adaptive cooperative guidance commands. If there are multiple models that have the same maximum probability, then we choose an arbitrary one to generate the adaptive cooperative guidance laws. For example, the simple way is to always choose the smallest value of *j* when there are multiple values.

In [15], the authors used the minimum mean-square-error criterion to generate the adaptive guidance commands, which are formulated as

(52) $$u_{TN}^{A}\left(k\right)=\sum_{j=1}^{N}\mu^{j}\left(k\right)u_{TN}^{*j}\left(k\right),\;\;u_{DN}^{A}=\sum_{j=1}^{N}\mu^{j}\left(k\right)u_{DN}^{*j}\left(k\right)$$

where the adaptive guidance commands are weighted sums of all model-matched guidance commands. According to Equation (52), at each guidance time instant, each model-matched cooperative guidance law needs calculating, and the calculation burden is a little heavy. The advantage of using the criterion of maximum a posteriori probability is that only one model-matched optimal cooperative guidance law needs computing at each guidance time instant. Thus, this will reduce the computational burden.

**Figure 3:**
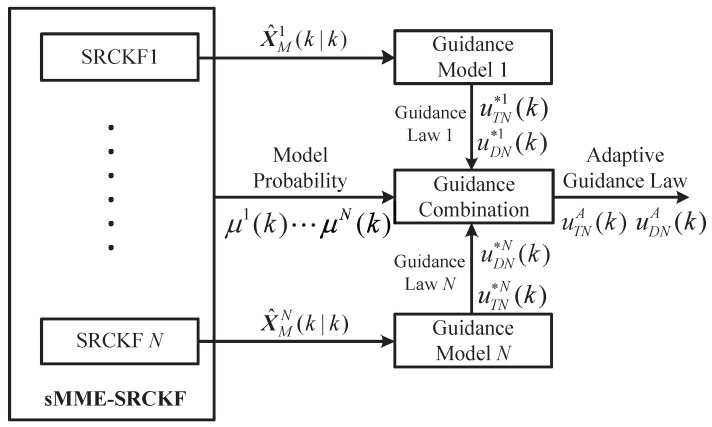
The structure of adaptive cooperative guidance laws.

The structure of adaptive cooperative guidance laws in the framework of the sMME-SRCKF is shown in Figure 3, where the output of the sMME-SRCKF (i.e., model-conditioned state estimation ${\hat{X}}_{M}^{j}\left(k|k\right)$ and model probability $\mu^{j}\left(k\right)$) is used to generate the adaptive guidance law. In Figure 3, guidance models 1–*N* represent *N* models based on Kinematic Equations (7)–(10) with the associated guidance law of the attacking missile. On the basis of the guidance model, the matched cooperative guidance laws (i.e., $u_{TN}^{*j}\left(k\right)$ and $u_{DN}^{*j}\left(k\right)$) are obtained by using Equation (44). The adaptive cooperative guidance laws are obtained by combining the model-matched cooperative guidance laws in the criterion of maximum a posteriori probability.

## 5. Simulations

In this section, the performance of the adaptive cooperative guidance laws and estimation approach is analyzed. The simulation conditions are as follows. The initial positions of three aircraft are $\left(x_{M0},y_{M0}\right)=\left(0,0\right)\;m$, $\left(x_{T0},y_{T0}\right)=\left(8000,0\right)\;m$, and $\left(x_{D0},y_{D0}\right)=\left(8000,0\right)\;m$; and the velocities are $V_{M}=700\;m/s$, $V_{T}=350\;m/s$, and $V_{D}=450\;m/s$. It is assumed that the three aircraft have first-order dynamics with time constants of $\tau_{M}=0.4\;s$, $\tau_{D}=0.4\;s$, and $\tau_{T}=0.1\;s$. The accelerations of the attacking missile, defender, and target are limited to $150\;m/s^{2}$, $120\;m/s^{2}$, and $80\;m/s^{2}$, respectively. The penalty weights in the cost function are set as $\alpha =10^{6}$ and β=1. The control efforts of the three aircraft are defined as $CE_{i}=\int_{0}^{t_{f_{MT}}}\left|u_{iN}\left(\tau \right)\right|d\tau$, with i=M,T,D. It is assumed that the maximum miss distance for successful interception is 5 m, so the defender or attacking missile will fail to intercept their targets beyond this range. Thus, the condition for successful active defense is $r_{MD}\left(t_{f_{MD}}\right)<5\;m$ or $r_{MT}\left(t_{f_{MT}}\right)>5\;m$.

### 5.1. Optimal Cooperative Guidance with Perfect Information

**Figure 4:**
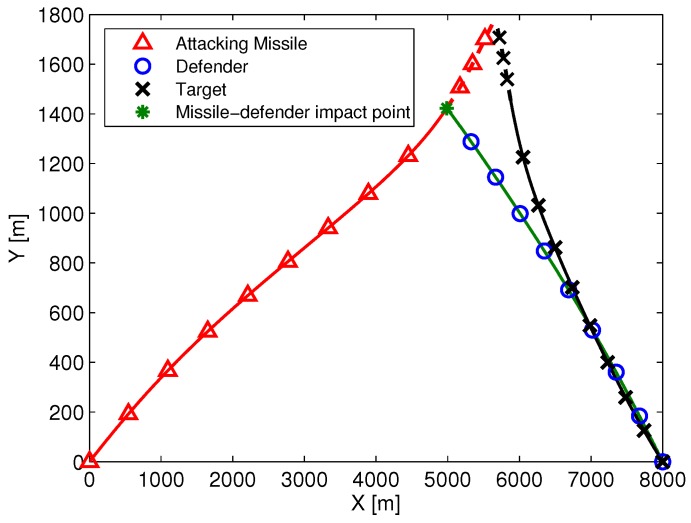
The trajectories of three aircraft in the case of $\left[\gamma_{M0},\gamma_{T0},\gamma_{D0}\right]=\left[20^{\circ },25^{\circ },30^{\circ }\right]$.

**Figure 5:**
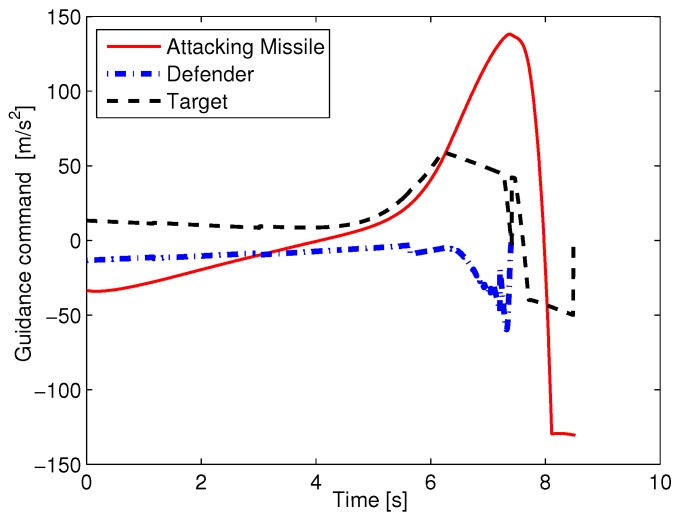
The guidance commands of three aircraft in the case of $\left[\gamma_{M0},\gamma_{T0},\gamma_{D0}\right]=\left[20^{\circ },25^{\circ },30^{\circ }\right]$.

In this subsection, the performance of model-matched optimal cooperative guidance with perfect information is tested. The attacking missile uses the proportional navigation (PN) guidance law, and $N_{\mathrm{PN}}=3$. First, the performance of optimal cooperative guidance laws with different initial flight-path angles is discussed. The initial flight-path angle of the attacking missile is set as $\gamma_{M0}=20^{\circ }$, and the initial flight-path angles of the defender and target are obtained from the sets $\left\{\gamma_{D0}\right\}=\left\{25^{\circ },30^{\circ }\right\}$ and $\left\{\gamma_{T0}\right\}=\left\{20^{\circ },25^{\circ },30^{\circ }\right\}$, respectively. The minimum expected evasion distance is set as ρ=10m. In the simulation, although the defender intercepts the attacking missile at the end of D–M engagement, we continue simulating the M–T engagement until it is completed in order to see the results of M–T engagement. The simulation results are shown in Table 1 and Figure 4 and Figure 5. In Table 1, we see that the D–M miss distances all approach zero, which demonstrates that the defender intercepts the attacking missile accurately. The M–T miss distance is almost equal to or a little larger than 10 m, which illustrates that the target achieves the expected minimum evasion distance. The reason that all of the M–T miss distances are close to the expected minimum evasion distance (i.e., 10 m) is as follows. After D–M engagement terminates, according to the evasion guidance law shown in Equation (47), if $Z_{MT}\left(t\right)\geq \rho$, then the maneuver of the target becomes zero, which makes $Z_{MT}\left(t\right)$ decrease; after $Z_{MT}\left(t\right)<\rho$, then the defender will execute an evasion maneuver to increase $Z_{MT}\left(t\right)$ until $Z_{MT}\left(t\right)\geq \rho$ again. Thus, $Z_{MT}\left(t\right)$ oscillates around ρ within a very small range, and the resultant M–T miss distances are close to ρ. Figure 4 and Figure 5 give the trajectories and guidance commands of the three aircraft in the case of $\left[\gamma_{M0},\gamma_{T0},\gamma_{D0}\right]=\left[20^{\circ },25^{\circ },30^{\circ }\right]$. In Figure 4, the solid lines represent the trajectories during the D–M engagement, and the dotted lines represent the trajectories after the D–M engagement. In Figure 4, the defender intercepts the attacking missile at the end of D–M engagement, and meanwhile, the target evades the attacking missile at the end of M–T engagement. In Figure 5, the guidance commands refer to $u_{iN}\;\left(i=T,D,M\right)$. The defender’s guidance command terminates at about 7.41 s because the D–M engagement terminates at that time. In the first 5.5 s, the guidance command of the target is small, and then the pursuit guidance command of the attacking missile is also small. As a result, the defender uses a small guidance command to pursue the attacking missile. After that, the target employs a larger evasion maneuver; then, the guidance command of the attacking missile increases, and this makes the defender use an aggressive maneuver to intercept the attacking missile.

**Figure 6:**
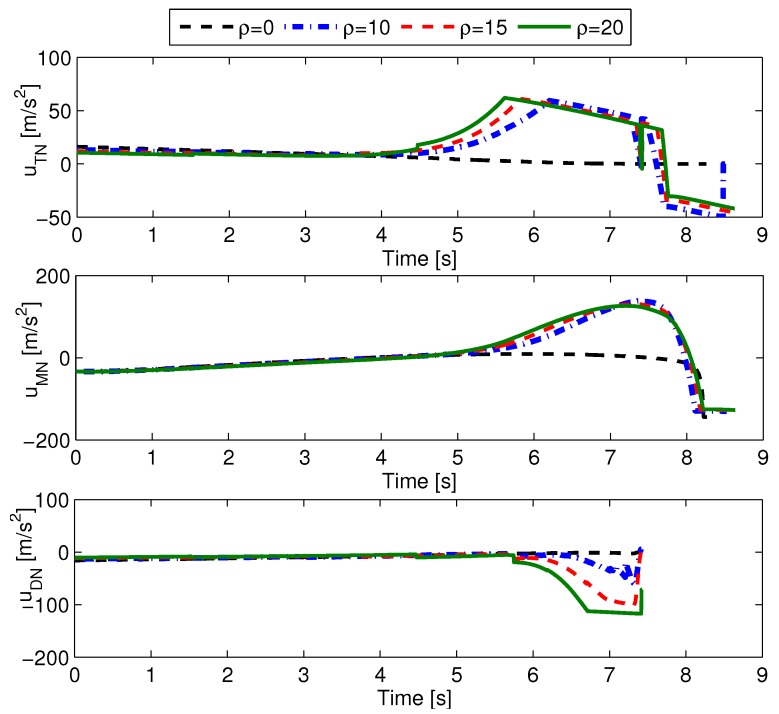
Guidance commands of three aircraft with different ρ.

Next, the performance of optimal cooperative guidance laws with different minimum evasion distances is presented. The minimum evasion distances are set as {ρ}={0,10,15,20}, and the initial flight-path angles are $\left[\gamma_{M0},\gamma_{T0},\gamma_{D0}\right]=\left[20^{\circ },25^{\circ },30^{\circ }\right]$. The simulation results are shown in Table 2 and Figure 6. In Table 2, $r_{MD}\left(t_{f_{MD}}\right)$ and $r_{MT}\left(t_{f_{MT}}\right)$ represent the D–M and M–T miss distances. From Table 2, we see that the larger the value of ρ, the more control effort each aircraft needs. Also, except for the case of ρ=20m, for which the D–M miss distance is a little large, all the other D–M miss distances are almost equal to zero. In the cases of ρ=15m and ρ=20m, the M–T miss distances are smaller than the expected minimum evasion miss distances. These results can be explained by Figure 6. In Figure 6, it is seen that the larger the value of ρ, the more aggressive the evasion maneuver used by the target uses, and as a consequence, the attacking missile and defender need more control effort to pursue their targets. Also, it is seen that the defender suffers command saturation for the longest time period in the case of ρ=20m, and that leads to a somewhat large D–M miss distance. Also, the target suffers relatively severe guidance command saturation in the case of ρ=15m and ρ=20m, and as a result, the target fails to reach the expected evasion miss distance. According to Table 2 and Figure 6, guidance command saturation is an important factor that influences the results of cooperative guidance laws.

In addition, there is another important factor that has a great impact on guidance performance via numerous simulations: the time constant of first-order dynamics. For example, increasing the time constant of the attacking missile to $\tau_{M}=0.6$ s, and keeping the other parameters the same, then in the case of ρ=20m, the simulation results are $r_{MD}\left(t_{f_{MD}}\right)=0.0388\;m$ and $r_{MT}\left(t_{f_{MT}}\right)=26.24\;m$. This example demonstrates that the defender can intercept the attacking missile accurately while the target meets the requirement for evasion miss distance. This is because the attacking missile becomes slow to respond to the guidance command in the case of a large time constant, and thus, the defender easily intercepts the attacking missile while the agile target is able to evade the attacking missile easily.

As a conclusion, guidance command limits and time constants both have an influence on the performance of optimal cooperative guidance laws. Here, we suggest setting the parameter ρ in cooperative guidance as follows: if the command limits of the defender and target are much larger than the attacking missile, or if the time constants of the target are much smaller than that of the attacking missile, then we can choose a large ρ to achieve both accurate defensive interception and large evasion miss distance. Otherwise, we need to choose a small ρ or even ρ=0 to focus on achieving accurate missile–defender miss distance. Additionally, according to the simulation results, the advantage of cooperative guidance laws is that the defender can intercept the maneuverable attacking missile with relatively smaller control effort with the help of the target. This advantage stems from the fact that the target employs a "lure" maneuver so that the attacking missile flies toward the defender.

### 5.2. Adaptive Cooperative Guidance Laws

Two adaptive cooperative guidance laws are defined, i.e., ACGL1 and ACGL2, and the expected minimum evasion distances in ACGL1 and ACGL2 are set as ρ=10m and ρ=0m, respectively. According to Remark 1, ACGL2 can be regarded as the adaptive guidance law that only considers a small D–M miss distance, which is identical to the cooperative guidance law considered in [2]. ACGL1 is the proposed cooperative guidance that considers both successful conditions, namely, a small D–M miss distance and the minimum M–T evasion distance.

The simulation conditions are as follows: the attacking missile uses the PN guidance law with $N_{\mathrm{PN}}=3$, and the initial flight path angles are $\left[\gamma_{M0},\gamma_{T0},\gamma_{D0}\right]=\left[20^{\circ },25^{\circ },30^{\circ }\right]$; both the measurement sampling period and guidance command period are 0.02 s, and the blind range is 500 m. The blind range refers to the minimal measuring range. When the defender approaches the attacking missile with a distance of less than 500 m, then the measurement model changes from Equations (13) to (15). The initial condition of the filter is sampled from a Gaussian distribution, i.e., ${\hat{X}}_{M}\left(0|0\right)∼N\left(X_{M}\left(0\right),P_{0}\right)$, where $X_{M}\left(0\right)$ is the true initial state of the attacking missile, and $P_{0}=\mathrm{diag}\left\{400^{2},400^{2},{\left(3\pi /180\right)}^{2},10^{2},20^{2}\right\}$ is the initial covariance.

First, the stochastic case in which measurement noise exists, and the attacking missile’s guidance law is known, is considered. In this case, the number of models in the sMME-SRCKF is 1, and thus, the filter becomes the SRCKF. Four cases with different measurement noises are considered—Case 1: $\sigma_{MT}\left(k\right)=\sigma_{MD}\left(k\right)=0.005\;\mathrm{rad}$; Case 2: $\sigma_{MT}\left(k\right)=\sigma_{MD}\left(k\right)=0.02\;\mathrm{rad}$; Case 3: $\sigma_{MT}\left(k\right)=\sigma_{MD}\left(k\right)=0.05\;\mathrm{rad}$; and Case 4: $\sigma_{MT}\left(k\right)=\sigma_{MD}\left(k\right)=0.08\;\mathrm{rad}$. The success probability of ACGL1 and ACGL2 are shown in Table 3. From Table 3, it is seen that if the measurement noise is small (i.e., Case 1), the success probabilities of ACGL1 and ACGL2 are both 100%. However, as the measurement noise increases, the success probability of ACGL1 becomes larger than that of ACGL2. When the measurement noise increases, it generates a larger estimation error and, as a result, a larger error in the guidance commands. ACGL2 only considers the accurate interception by the defender. Thus, once the defender misses the attacking missile because of guidance error, the active defense fails. On the contrary, for ACGL1, the target will take evasive measures after the failed D–M engagement, thus increasing the probability of success. This is the advantage of ACGL1 that can lead to a better performance in a noisy environment compared with ACGL2.

**Figure 7:**
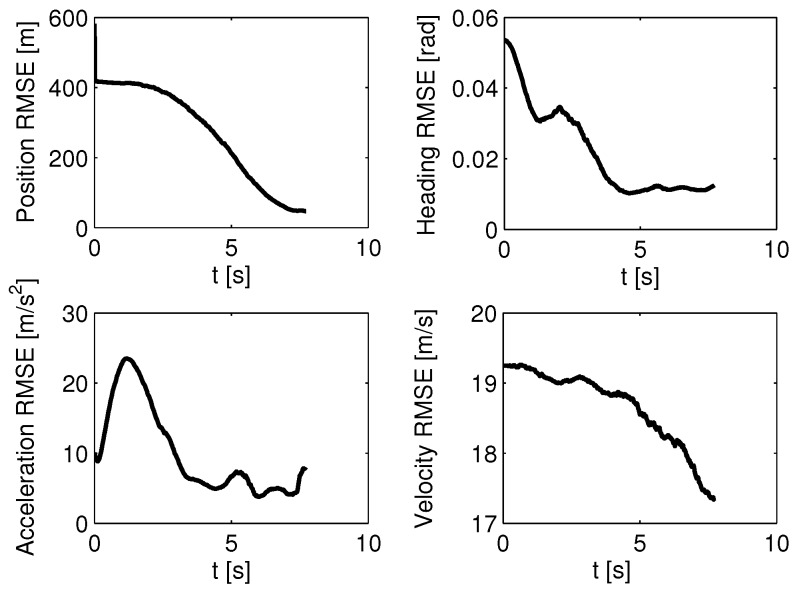
Estimation error of the sMME-SRCKF (static multiple model estimator with square-root cubature Kalman filter) in Case 1.

**Figure 8:**
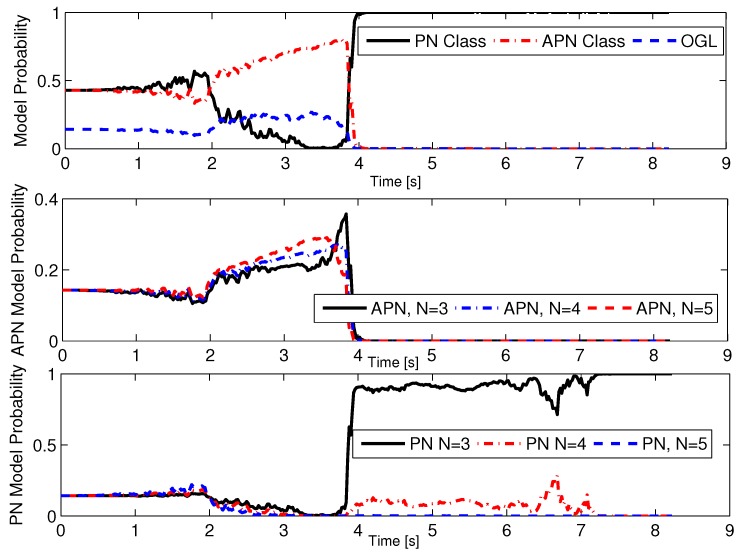
Model probability of the sMME-SRCKF for one Monte Carlo simulation in Case 1.

Next, another stochastic case is considered: the attacking missile’s guidance strategy is unknown, and we use the sMME-SRCKF to identify the guidance strategy. The set of guidance laws contains PN with $N_{\mathrm{PN}}=3,4,5$, APN with $N_{\mathrm{APN}}=3,4,5$, and OGL. The initial probability of each guidance model is 1/7. The actual guidance law of the attacking missile is PN with $N_{\mathrm{PN}}=3$. Three cases of measurement noise are set as follows. Case 1: $\sigma_{MT}\left(k\right)=\sigma_{MD}\left(k\right)=0.02\;\mathrm{rad}$; Case 2: $\sigma_{MT}\left(k\right)=\sigma_{MD}\left(k\right)=0.05\;\mathrm{rad}$; and Case 3: $\sigma_{MT}\left(k\right)=\sigma_{MD}\left(k\right)=0.08\;\mathrm{rad}$. For each case, a 1000-run Monte Carlo simulation was completed. The success probability of ACGL1 in Case 1, Case 2, and Case 3 is 86.6%, 67.8%, and 57.2%, respectively; that of ACGL2 in Case 1, Case 2, and Case 3 is 84.5%, 47.1%, and 36.1%, respectively. These results show that ACGL1 still performs better than ACGL2. The simulation results of ACGL1 in Case 1 are shown in Figure 7 and Figure 8. Figure 7 shows the root-mean-square error (RMSE) of the estimator. The RMSE of the estimated scalar $\hat{x}$ is defined as [16]

(53) $$\mathrm{RMSE}\left(\hat{x}\right)=\sqrt{\frac{\sum_{i=1}^{N_{MC}}{\left(x-{\hat{x}}_{i}\right)}^{2}}{N_{MC}}}$$

where $N_{MC}$ represents the number of Monte Carlo simulations. For example, the position RMSE is defined as

(54) $$\mathrm{RMSE}\left(\mathrm{Pos}\right)=\sqrt{\frac{1}{N_{MC}}\sum_{i=1}^{N_{MC}}{\left(x_{M}-{\hat{x}}_{M,i}\right)}^{2}+{\left(y_{M}-{\hat{y}}_{M,i}\right)}^{2}}$$

where $\left({\hat{x}}_{M,i},{\hat{y}}_{M,i}\right)$ is the estimated position for the *i*th Monte Carlo simulation. In Figure 7, each RMSE converges as time moves forward, which demonstrates that the sMME-SRCKF works well. In Figure 8, the model probability of the sMME-SRCKF in a single simulation is presented, and the PN class refers to PN guidance laws with $N_{\mathrm{PN}}=3,4,5$, and the APN class refers to APN guidance laws with $N_{\mathrm{APN}}=3,4,5$. Figure 8 shows that the model probability of the PN class increases to 1, and the other model’s probability reduces to zero at about 4 s. The sMME-SRCKF can be assumed to identify the correct guidance law of the attacking missile at about 4 s, since the model probability of PN with $N_{\mathrm{PN}}=3$ plays a dominant role, i.e., the model probability of PN with $N_{\mathrm{PN}}=3$ is larger than 85% for the most time from 4 s to the end.

As a conclusion, it is demonstrated that the proposed adaptive guidance law that considers two successful conditions has a larger probability of a successful active defense compared with the adaptive guidance law that only considers one successful condition.

### 5.3. Estimation Performance Evaluation

#### 5.3.1. Comparison of Filtering Approaches

**Figure 9:**
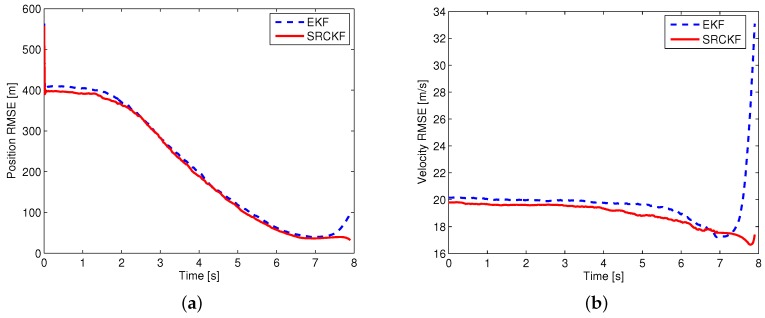
Estimation errors of the extended Kalman filter (EKF) and SRCKF: (**a**) position root-mean-square error (RMSE), (**b**) velocity RMSE.

**Figure 10:**
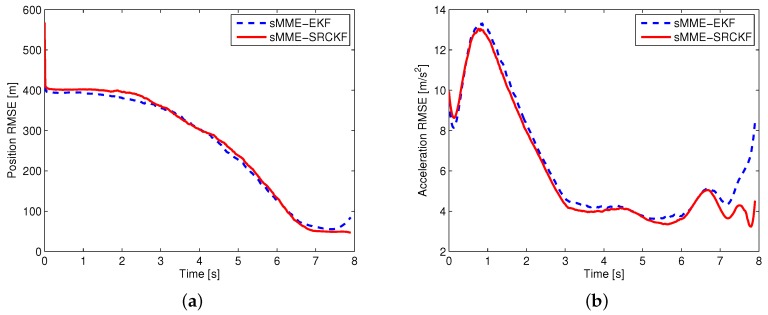
Estimation errors of the sMME-EKF and sMME-SRCKF: (**a**) position RMSE, (**b**) acceleration RMSE.

For a fair comparison of different filtering approaches, perfect information is used for calculating the cooperative guidance laws. Using this approach, the performance evaluation of the filter is separated from the closed-loop system of guidance and estimation. Two scenarios are simulated for evaluating the performance of different filtering approaches. In the first scenario, it is assumed that the guidance law of the attacking missile is known, and the measurement noise is $\sigma_{MT}\left(k\right)=\sigma_{MD}\left(k\right)=0.01\;\mathrm{rad}$. Then, the EKF and SRCKF are used to estimate the state of the attacking missile. In the second scenario, it is assumed that the guidance law of the attacking missile is unknown, and the measurement noise is $\sigma_{MT}\left(k\right)=\sigma_{MD}\left(k\right)=0.02\;\mathrm{rad}$. The sMME-EKF and sMME-SRCKF are used to track the attacking missile. Except for the measurement noise, the simulation conditions of the two scenarios are the same as those shown in Section 5.2. The attacking missile uses PN with $N_{PN}=3$, and the expected minimum evasion distance of the cooperative guidance laws is set as ρ=10. As indicated in Section 3.1, the complex Jacobian matrix needs computing to implement the EKF and sMME-EKF. For the sake of brief exposition, the derivation of the Jacobian matrix is omitted here, and the process of the sMME-EKF can be referred to [15]. A 1000-run Monte Carlo simulation was performed in both scenarios, and the simulation results are shown in Figure 9 and Figure 10.

According to the simulation results, D–M engagement is terminated at 6.94 s, and M–T engagement is terminated at 7.9 s. Note that during D–M engagement, two sensors are used to track the attacking missile; after the termination of D–M engagement, only a sensor on the target works to track the attacking missile. From Figure 9, it is seen that the performance of the SRCKF is a little better than that of EKF during the D–M engagement (i.e., 0–6.94 s); after that, the performance of the SRCKF is much better than that of EKF (i.e., 6.94–7.9 s). In Figure 10, it is seen that the performance of the sMME-SRCKF is almost the same as that of the sMME-EKF during the D–M engagement (i.e., 0–6.94 s), while the sMME-SRCKF performs better than the sMME-EKF after the termination of D–M engagement (i.e., 6.94–7.9 s). The simulation results demonstrate the superiority of the SRCKF or sMME-SRCKF, especially after the termination of D–M engagement. This is because only one sensor with angular measurement is used to track the attacking missile at this phase, and then the nonlinearity of the estimation problem becomes more serious. Furthermore, the SRCKF and sMME-SRCKF are derivative-free for undesirable Jacobians and the transition matrix. It is convenient to implement the SRCKF and sMME-SRCKF for various guidance laws of attacking missile. For example, if a new guidance law is added to the sMME filter, then the additional derivation of the Jacobian matrix and calculation of the transition matrix are needed for the sMME-EKF. However, this is not required in the sMME-SRCKF.

#### 5.3.2. Estimation Enhancement Test

**Figure 11:**
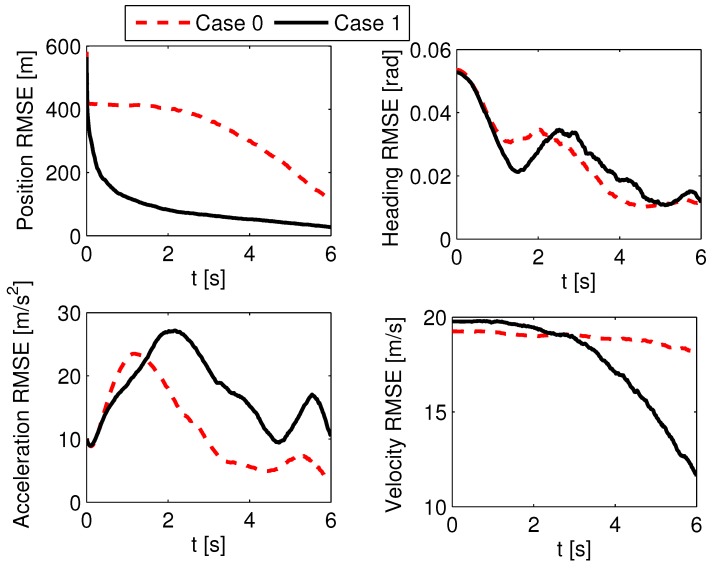
Estimation RMSE of the sMME-SRCKF in Case 0 and Case 1.

**Figure 12:**
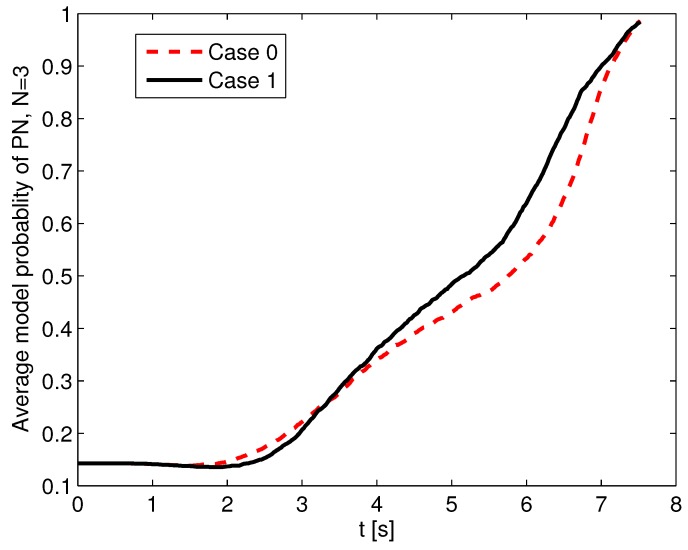
Average model probability of PN with $N_{\mathrm{PN}}=3$ in Case 0 and Case 1.

According to the analysis in Section 3.2, it is concluded that the LOS angle difference will influence the estimation performance. Thus, the estimation performance is tested with different initial LOS angles. Here, two cases are compared. In Case 0, all the initial conditions are as shown at the beginning of Section 5. In Case 1, the initial position and flight-path angle of the defender are changed to $\left(x_{D0},y_{D0}\right)=\left(8000,2000\right)\;m$ and $\gamma_{D0}=-15^{\circ }$, and the rest of the initial conditions are the same as those of Case 0. The absolute initial LOS angle differences in Case 0 and Case 1 are $0^{\circ }$ and $14^{\circ }$, respectively. The stochastic scenario of an unknown guidance strategy of the attacking missile is considered. The set of guidance laws contains PN with $N_{\mathrm{PN}}=3,4,5$, APN with $N_{\mathrm{APN}}=3,4,5$, and OGL, and the initial probability of each guidance model is set as 1/7. The measurement noise is $\sigma_{MT}\left(k\right)=\sigma_{MD}\left(k\right)=0.02\;\mathrm{rad}$, and the attacking missile uses the PN guidance law with $N_{\mathrm{PN}}=3$. ACGL1 in Section 5.2 is used as the adaptive guidance law. A 1000-run Monte Carlo simulation was completed, and the probability of success in Case 1 is 91.2%, which is better than that in Case 0 (i.e., 86.6%). Also, the probability of $r_{MD}\left(t_{f_{MD}}\right)<5\;m$ in Case 1 is 68.9%, which is larger than that of 63.1% in Case 0. The increased success probability in Case 1 benefits from the estimation enhancement, whose results are shown in Figure 11 and Figure 12. Figure 11 shows the RMSEs of position, flight-path angle, acceleration, and velocity in the first 6 s. It shows that the RMSEs of position and velocity in Case 1 converge more rapidly than those in Case 0. The position RMSE in Case 1 at 6 s is 27 m, which is much smaller than that of 115 m in Case 0. The RMSEs of flight-path angle and acceleration in Case 0 and Case 1 perform in a similar way. In Figure 12, the average model probabilities of PN with $N_{\mathrm{PN}}=3$ in Case 0 and Case 1 are shown. The average model probability is introduced as an index to represent the change in model probability in the Monte Carlo simulation, and it is more reliable to use this index than to use the model probability in a single Monte Carlo simulation. The average model probability of the *j*th model ${\bar{u}}^{j}\left(k\right)$ is defined as

(55) $${\bar{u}}^{j}\left(k\right)=\frac{1}{N_{MC}}\sum_{i=1}^{N_{MC}}u_{i}^{j}\left(k\right)$$

where $u_{i}^{j}\left(k\right)$ is the *j*th model probability at the *i*th MC simulation, and $N_{MC}$ is the number of Monte Carlo simulations. From Figure 12, it is seen that the average model probability of PN guidance law with $N_{\mathrm{PN}}=3$ in Case 1 is always larger than that in Case 0 after 4 s, so the sMME-SRCKF in Case 1 can identify the right guidance strategy faster on the average. The faster the sMME-SRCKF identifies the right model, the more accurate the generated guidance command, and the larger the probability of a successful active defense. Thus, it is helpful to choose an engagement geometry with a large initial LOS angle difference to yield good estimation and guidance performance.

## 6. Conclusions

In this paper, adaptive cooperative guidance for a target and defender is proposed to deal with the stochastic active defense problem. Adaptive cooperative guidance combines a multiple-model adaptive estimator and optimal control. The sMME-SRCKF is designed as a nonlinear adaptive estimator that can identify the guidance strategy and estimate the state of the attacking missile efficiently. By solving the optimal defensive problem, the model-matched cooperative guidance laws are obtained that can satisfy criteria of both an accurate defensive interception and the expected minimum evasion distance. The cooperation between the target and defender is established by using the cooperative guidance laws, and the advantage of this cooperation makes it possible to use a low-maneuverability defending missile (the cost of this low-maneuverability missile is cheap) to intercept an advanced and high-maneuverability attacking missile. Also, the adaptive cooperative guidance law performs better in the stochastic scenario, and it is more robust than the adaptive guidance law that only considers small D–M miss distance. Furthermore, the estimation enhancement analysis provides an approach to improving the performance of the estimation and guidance.

This paper focuses on the design of cooperative guidance laws in planar active defense engagement. For the general three-dimensional active defense engagement, it can be decoupled into two perpendicular planar engagements, and then the proposed guidance laws can be applied to both planar engagements. Further work lies in extending this proposed solution to three-dimensional engagement.

## Figures and Tables

**Table 1:** Simulation results with different flight-path angles. D—defender; M—missile; T—target.

$\left[\gamma_{M0},\gamma_{T0},\gamma_{D0}\right]$	D–M Miss Distance (m)	M–T Miss Distance (m)
$\left[20^{\circ },20^{\circ },25^{\circ }\right]$	0.0022	10.3023
$\left[20^{\circ },20^{\circ },30^{\circ }\right]$	0.0027	11.5965
$\left[20^{\circ },25^{\circ },25^{\circ }\right]$	0.006	10.7112
$\left[20^{\circ },25^{\circ },30^{\circ }\right]$	0.0017	11.1289
$\left[20^{\circ },30^{\circ },25^{\circ }\right]$	0.0014	11.0299
$\left[20^{\circ },30^{\circ },30^{\circ }\right]$	0.0164	10.4282

**Table 2:** Simulation results in different cases.

Simulation Case	$r_{\mathrm{MD}}\left(t_{f_{\mathrm{MD}}}\right)$ (m)	$r_{\mathrm{MT}}\left(t_{f_{\mathrm{MT}}}\right)$ (m)	${\mathrm{CE}}_{M}$ (m/s)	${\mathrm{CE}}_{T}$ (m/s)	${\mathrm{CE}}_{D}$ (m/s)
ρ=0 m	0.0031	0.071	109.6531	56.8811	59.8561
ρ=10 m	0.0017	11.1289	354.9131	193.5426	81.5393
ρ=15 m	0.0619	10.2815	379.1928	204.7415	129.7482
ρ=20 m	2.7552	9.5650	393.9062	208.7452	173.0856

**Table 3:** Successful probability of two adaptive cooperative guidance laws (ACGL) with different measurement noise.

	Case 1	Case 2	Case 3	Case 4
ACGL1	100%	99.9%	93.9%	83.1%
ACGL2	100%	88%	57.5%	47.4%

## Abbreviation

A, $A_{11}$, $A_{12}$, $A_{21}$, $A_{22}$, $B_{T}$, $B_{D}$	state-space model matrices of active defense
$A_{i}$, $b_{i}$, $C_{i}$, $d_{i}$	state-space model matrices of aircraft’s internal dynamics
$a_{M}$, $a_{T}$, $a_{D}$	acceleration of attacking missile, target, and defender
$a_{MN}$, $a_{TN}$, $a_{DN}$	acceleration component of attacking missile, target, and defender along the *Y*-axis
$k_{1}$, $k_{2}$, $k_{M}$, $k_{T}$, $k_{uT}$	guidance parameters of the attacking missile
$N_{PN}$, $N_{APN}$, $N_{OGL}$	navigation gains of PN, APN, and OGL
$n_{M}$, $n_{T}$, $n_{D}$	dimension of the internal state vector of the attacking missile, target, and defender
$q_{M}$, $q_{T}$, $q_{D}$	internal state vector of the attacking missile, target, and defender
$R_{k}$	measurement noise covariance matrix at time instant *k*
$t_{f_{MD}}$, $t_{f_{MT}}$, $t_{go_{MT}}$	D–M and M–T interception time, time-to-go of M–T engagement
$u_{M}$, $u_{T}$, $u_{D}$	guidance command of the attacking missile, target, and defender
$u_{MN}$, $u_{TN}$, $u_{DN}$	guidance command of the attacking missile, target, and defender along the *Y*-axis
$u_{TN}^{A}$, $u_{DN}^{A}$	adaptive guidance laws of the target and defender
X, $X_{M}$	state vector of active defense kinematic model and state vector of the attacking missile
${\hat{X}}^{j}$	*j*th model-conditioned state estimate of active defense
${\hat{X}}_{M}\left(k|k\right)$, P(k|k)	state estimate and error covariance of attacking missile
${\hat{X}}_{M}^{j}\left(k|k\right)$, $P^{j}\left(k|k\right)$	*j*th model-conditioned state estimate and error covariance of the attacking missile
$y_{MD}$, $y_{MT}$, ${\dot{y}}_{MD}$, ${\dot{y}}_{MT}$	D–M and M–T relative displacements and relative velocity along the *Y*-axis
$Z_{MD}$, $Z_{MT}$	D–M and M–T zero-effort miss distances
z(k), $z_{MD}\left(k\right)$, $z_{MT}\left(k\right)$	measurement vector, D–M and M–T angle measurement at time instant k
${\tilde{z}}^{j}\left(k|k\right)$, $P_{zz}^{j}\left(k|k-1\right)$	*j*th model-conditioned innovation and innovation covariance at time instant k
α, β	penalty weights in the cost function
$\gamma_{M}$, $\gamma_{T}$, $\gamma_{D}$	flight-path angle of the attacking missile, target, and defender
$\Lambda^{j}\left(k\right)$, $\mu^{j}\left(k\right)$	jth model-conditioned likelihood function and *j*th model probability
$\lambda_{MD}$, $\lambda_{MT}$	D–M and M–T line-of-sight angle
ν, $\nu_{MD}$, $\nu_{MT}$	white Gaussian noise vector, D–M and M–T angle measurement noise
ρ, Φ	expected evasion miss distance, transition matrix
$\tau_{M}$, $\tau_{T}$, $\tau_{D}$	time constant of the attacking missile, target and defender

## Appendix A.

The algorithm for using the SRCKF to estimate the model-conditioned state of the attacking missile is shown as follows. The variables with the subscript *j* represent the variables of the *j*th model. According to [17], the SRCKF is described by two steps: time update and measurement update.

In the time update, the cubature points are firstly evaluated:

(A1) $$X_{M,i}^{j}\left(k-1|k-1\right)=S^{j}\left(k-1|k-1\right)\xi_{i}+{\hat{X}}_{M}^{j}\left(k-1|k-1\right)$$

with (i=1,2,⋯,m), where $m=2n_{x}$ is the number of cubature points, and $n_{x}$ represents the dimension of $X_{M}^{j}$. ${\hat{X}}_{M}^{j}\left(k-1|k-1\right)$ and $S^{j}\left(k-1|k-1\right)$ are the attacking missile state estimate and the square-root factor of error covariance at the k−1 time instant; $\xi_{i}=\sqrt{\frac{m}{2}}{\left[1\right]}_{i}$, where ${\left[1\right]}_{i}$ is the *i*th point/column of the complete fully symmetric set of points defined as $\left[1\right]=\left[I_{n_{M}},-I_{n_{M}}\right]$. The process equation in this section is a continuous differential equation rather than a difference equation as shown in [17]. Thus, according to Equation (12), the evaluation of the propagated cubature points in this section can be calculated as

(A2) $$X_{M,i}^{j*}\left(k|k-1\right)=X_{M,i}^{j}\left(k-1|k-1\right)+\int_{(k-1)T}^{kT}f(X_{M,i}^{j}\left(t\right),\;u_{MN}^{j}\left(\left(k-1\right)T\right))dt$$

with (i=1,2,⋯,m), where $X_{M,i}^{j}\left(t\right)$ is the *i*th cubature point at time *t*, and *T* is the sampling period. The integration in Equation (A2) can be computed by using the fourth-order Runge–Kutta method. Then, the predicted state and square-root factor of the predicted error covariance are

(A3) $${\hat{X}}_{M}^{j}\left(k|k-1\right)=\frac{1}{m}\sum_{i=1}^{m}X_{M,i}^{j*}\left(k|k-1\right)$$

(A4) $$S^{j}\left(k|k-1\right)=\mathrm{Tria}\left([\chi^{j*}\left(k|k-1\right),\;\;S_{Q}\left(k-1\right)]\right)$$

where

(A5) $$\begin{array}{cc} \chi^{j*}\left(k|k-1\right)=\frac{1}{\sqrt{m}}[ & X_{M,1}^{j*}\left(k|k-1\right)-{\hat{X}}_{M}^{j}\left(k|k-1\right),X_{M,2}^{j*}\left(k|k-1\right)-{\hat{X}}_{M}^{j}\left(k|k-1\right),\cdots ,\\ & X_{M,m}^{j*}\left(k|k-1\right)-{\hat{X}}_{M}^{j}\left(k|k-1\right)] \end{array}$$

and $S_{Q}\left(k-1\right)$ is a square-root of the covariance of process noise satisfying $S_{Q}\left(k-1\right)S_{Q}^{T}\left(k-1\right)=Q\left(k-1\right)$ with Q(k−1) representing the covariance of process noise. According to Equation (11), we have Q(k−1)=0, which means that there is no process noise. Tria(•) represents a general triangularization algorithm, and we have $\mathrm{Tria}(A)=R^{T}$, where R is an upper triangular matrix obtained from the QR decomposition on $A^{T}$.

In the measurement update, the predicted measurement and the square root of the innovation covariance are firstly estimated:

(A6) $${\hat{z}}^{j}(k|k-1)=\frac{1}{m}\sum_{i=1}^{m}Z_{i}^{j}(k|k-1)$$

(A7) $$S_{zz}^{j}(k|k-1)=\mathrm{Tria}\left([Z^{j}(k|k-1),\;S_{R}\left(k\right)]\right)$$

where

(A8) $$Z^{j}\left(k|k-1\right)=\frac{1}{\sqrt{m}}\left[Z_{1}^{j}\left(k|k-1\right)-{\hat{z}}^{j}\left(k|k-1\right),Z_{2}^{j}\left(k|k-1\right)-{\hat{z}}^{j}\left(k|k-1\right),\cdots ,Z_{m}^{j}\left(k|k-1\right)-{\hat{z}}^{j}\left(k|k-1\right)\right]$$

and

(A9) $$Z_{i}^{j}(k|k-1)=h\left(X_{M,i}^{j}(k|k-1)\right)=h\left(S^{j}(k|k-1)\xi_{i}+{\hat{X}}_{M}^{j}(k|k-1)\right)$$

and $S_{R}\left(k\right)$ is the square-root factor of $R_{k}$ (i.e., measurement noise covariance) satisfying $S_{R}\left(k\right)S_{R}^{T}\left(k\right)=R\left(k\right)$. The innovation ${\tilde{z}}^{j}\left(k|k\right)$ and the innovation covariance are computed as

(A10) $$\begin{array}{cc} {\tilde{z}}^{j}\left(k|k\right) & =z\left(k\right)-{\hat{z}}^{j}(k|k-1)\\ P_{zz}^{j}\left(k|k-1\right) & =S_{zz}^{j}\left(k|k-1\right){\left(S_{zz}^{j}\left(k|k-1\right)\right)}^{T} \end{array}$$

Then, the updated state estimate ${\hat{X}}_{M}^{j}\left(k|k\right)$ and the square-root factor of the error covariance $S^{j}\left(k|k\right)$ are

(A11) $${\hat{X}}_{M}^{j}\left(k|k\right)={\hat{X}}_{M}^{j}\left(k|k-1\right)+W^{j}\left(k\right)\left(z(k)-{\hat{z}}^{j}\left(k|k-1\right)\right)$$

(A12) $$S^{j}\left(k|k\right)=Tria\left(\left[\chi^{j}\left(k|k-1\right)-W^{j}\left(k\right)Z^{j}\left(k|k-1\right),W^{j}\left(k\right)S_{R}^{j}\left(k\right)\right]\right)$$

where

(A13) $$\begin{array}{cc} \chi^{j}\left(k|k-1\right)=\frac{1}{\sqrt{m}}[ & X_{M,1}^{j}\left(k|k-1\right)-{\hat{X}}_{M}^{j}\left(k|k-1\right),X_{M,2}^{j}\left(k|k-1\right)-{\hat{X}}_{M}^{j}\left(k|k-1\right),\cdots ,\\ & X_{M,m}^{j}\left(k|k-1\right)-{\hat{X}}_{M}^{j}\left(k|k-1\right)] \end{array}$$

and

(A14) $$\begin{array}{c} W^{j}\left(k\right)=\left(P_{xz}^{j}\left(k|k-1\right)/{\left(S_{zz}^{j}\left(k|k-1\right)\right)}^{T}\right)/S_{zz}^{j}\left(k|k-1\right)\\ P_{xz}^{j}\left(k|k-1\right)=\chi^{j}\left(k|k-1\right){\left(Z^{j}\left(k|k-1\right)\right)}^{T} \end{array}$$

The error covariance of the estimation can be obtained as

(A15) $$P^{j}\left(k|k\right)=S^{j}\left(k|k\right){\left(S^{j}\left(k|k\right)\right)}^{T}$$

This completes the algorithm for using the SRCKF to estimate the model-conditioned state of the attacking missile.

## Appendix B.

The derivation of Equations (30) and (31) is shown in this appendix. Since Φ is the transition matrix associated with Equation (7), it has the following property:

(A16) $$\dot{\Phi }\left(t_{f},t\right)=-\Phi \left(t_{f},t\right)A\left(t\right);\;\Phi \left(t_{f},t_{f}\right)=I$$

Taking the derivative of Equation (28) and using Equations (7) and (A16), then we have

(A17) $$\left\{\begin{array}{c} \begin{array}{cc} {\dot{Z}}_{MT}\left(t\right) & =D_{MT}\Phi \left(t_{f_{MT}},t\right)\left(B_{T}u_{TN}+B_{D}u_{DN}\right),\;\;\;\;\;t\in \left[0,t_{f_{MT}}\right]\\ {\dot{Z}}_{MD}\left(t\right) & =\left\{\begin{array}{c} D_{MD}\Phi \left(t_{f_{MD}},t\right)\left(B_{T}u_{TN}+B_{D}u_{DN}\right),\;\;t\in \left[0,t_{f_{MD}}\right]\\ 0,\;t\in (t_{f_{MD}},t_{f_{MT}}] \end{array}\right. \end{array} \end{array}\right.$$

In Equation (A17), we have

(A18) $$D_{MT}\Phi \left(t_{f_{MT}},t\right)B_{D}=0$$

This can be proved by the following steps. Defining the first row of $\Phi (t_{f_{MT}},t)$ as $\left[\phi_{11},\phi_{12},\phi_{1M},\phi_{1T},\phi_{13},\phi_{14},\phi_{1D}\right]$, where $\dim(\phi_{1i})=1\times n_{i}$ for i=T,D,M, we therefore have

(A19) $$D_{MT}\Phi \left(t_{f_{MT}},t\right)B_{D}=-\phi_{14}d_{D}+\phi_{1D}b_{D}$$

According to Equation (A16), we have

(A20) $$\left\{\begin{array}{c} {\dot{\phi }}_{13}\left(t_{f_{MT}},t\right)=0,\;\;\phi_{13}\left(t_{f_{MT}},t_{f_{MT}}\right)=0\\ {\dot{\phi }}_{14}\left(t_{f_{MT}},t\right)=-\phi_{13}\left(t_{f_{MT}},t\right),\;\;\phi_{14}\left(t_{f_{MT}},t_{f_{MT}}\right)=0\\ {\dot{\phi }}_{1D}\left(t_{f_{MT}},t\right)=\phi_{14}\left(t_{f_{MT}},t\right)C_{D}-\phi_{1D}\left(t_{f_{MT}},t\right)A_{D},\;\;\phi_{1D}\left(t_{f_{MT}},t_{f_{MT}}\right)=0 \end{array}\right.$$

Then, on the basis of Equation (A20), the following equation is obtained:

(A21) $$\phi_{13}\left(t_{f_{MT}},t\right)\equiv 0,\;\;\phi_{14}\left(t_{f_{MT}},t\right)\equiv 0,\;\;\phi_{1D}\left(t_{f_{MT}},t\right)\equiv 0$$

Thus, by using Equations (A19) and (A21), Equation (A18) is obtained. Combining Equations (A17) and (A18), Equations (30) and (31) are obtained.

## Appendix C.

This appendix gives the proof of $L_{1}<0$. According to Equation (38), we have $F_{1}<0$ and

(A22) $$F_{2}^{2}-F_{1}F_{3}={\left(\int_{t}^{t_{f_{MT}}}\frac{f_{T_{MT}}\left(\tau \right)f_{T_{MD}}\left(\tau \right)}{\beta }d\tau \right)}^{2}-\int_{t}^{t_{f_{MT}}}\frac{f_{T_{MT}}^{2}\left(\tau \right)}{\beta }d\tau \cdot \int_{t}^{t_{f_{MT}}}\frac{f_{T_{MD}}^{2}\left(\tau \right)}{\beta }+f_{D_{MD}}^{2}\left(\tau \right)d\tau$$

First, the auxiliary inequality, i.e., $F_{2}^{2}-F_{1}F_{3}\leq 0$, is proved via the following steps. Assuming $\vec{a}=\left(\frac{f_{T_{MT}}}{\sqrt{\beta }},0\right)$ and $\vec{b}=\left(\frac{f_{T_{MD}}}{\sqrt{\beta }},f_{D_{MD}}\right)$, then we have $\vec{a}•\vec{b}=\frac{f_{T_{MT}}f_{T_{MD}}}{\beta }$. Since $\left|\vec{a}•\vec{b}\right|\leq \left|\vec{a}\right|\left|\vec{b}\right|$, the following equation is obtained.

(A23) $$\left|\frac{f_{T_{MT}}f_{T_{MD}}}{\beta }\right|\leq \sqrt{\frac{f_{T_{MT}}^{2}}{\beta }}\sqrt{\frac{f_{T_{MD}}^{2}}{\beta }+f_{D_{MD}}^{2}}$$

Integrating Equation (A23) from *t* to $t_{f_{MT}}$, we have

(A24) $$\int_{t}^{t_{f_{MT}}}\left|\frac{f_{T_{MT}}\left(\tau \right)f_{T_{MD}}\left(\tau \right)}{\beta }\right|d\tau \leq \int_{t}^{t_{f_{MT}}}\sqrt{\frac{f_{T_{MT}}^{2}\left(\tau \right)}{\beta }}\sqrt{\frac{f_{T_{MD}}^{2}\left(\tau \right)}{\beta }+f_{D_{MD}}^{2}\left(\tau \right)}d\tau$$

By using the Cauchy–Buniakowsky–Schwarz Inequality, the right side of Equation (A24) satisfies

(A25) $$\int_{t}^{t_{f_{MT}}}\sqrt{\frac{f_{T_{MT}}^{2}\left(\tau \right)}{\beta }}\sqrt{\frac{f_{T_{MD}}^{2}\left(\tau \right)}{\beta }+f_{D_{MD}}^{2}\left(\tau \right)}d\tau \leq \sqrt{\left(\int_{t}^{t_{f_{MT}}}\frac{f_{T_{MT}}^{2}\left(\tau \right)}{\beta }d\tau \cdot \int_{t}^{t_{f_{MT}}}\frac{f_{T_{MD}}^{2}\left(\tau \right)}{\beta }+f_{D_{MD}}^{2}\left(\tau \right)d\tau \right)}$$

On the basis of Equations (A24) and (A25) and using ${\left(\int_{t}^{t_{f_{MT}}}\frac{f_{T_{MT}}\left(\tau \right)f_{T_{MD}}\left(\tau \right)}{\beta }d\tau \right)}^{2}\leq {\left(\int_{t}^{t_{f_{MT}}}\left|\frac{f_{T_{MT}}\left(\tau \right)f_{T_{MD}}\left(\tau \right)}{\beta }\right|d\tau \right)}^{2}$, we have

(A26) $${\left(\int_{t}^{t_{f_{MT}}}\frac{f_{T_{MT}}\left(\tau \right)f_{T_{MD}}\left(\tau \right)}{\beta }d\tau \right)}^{2}\leq \int_{t}^{t_{f_{MT}}}\frac{f_{T_{MT}}^{2}\left(\tau \right)}{\beta }d\tau \cdot \int_{t}^{t_{f_{MT}}}\frac{f_{T_{MD}}^{2}\left(\tau \right)}{\beta }+f_{D_{MD}}^{2}\left(\tau \right)d\tau$$

Thus, $F_{2}^{2}-F_{1}F_{3}\leq 0$ is proved. By using $L_{1}=F_{1}-\alpha F_{1}F_{3}+\alpha F_{2}^{2}$, $F_{1}<0$, α>0, and $F_{2}^{2}-F_{1}F_{3}\leq 0$, then $L_{1}<0$ is proved.
